# Plant Physiological, Morphological and Yield-Related Responses to Night Temperature Changes across Different Species and Plant Functional Types

**DOI:** 10.3389/fpls.2016.01774

**Published:** 2016-11-24

**Authors:** Panpan Jing, Dan Wang, Chunwu Zhu, Jiquan Chen

**Affiliations:** ^1^International Center for Ecology, Meteorology, and Environment, School of Applied Meteorology, Nanjing University of Information Science and TechnologyNanjing, China; ^2^State Key Laboratory of Soil and Sustainable Agriculture, Institute of Soil Science, Chinese Academy of SciencesNanjing, China; ^3^CGCEO/Geography, Michigan State UniversityEast Lansing, MI, USA

**Keywords:** high night temperature, low night temperature, photosynthesis, respiration, biomass, plant functional types

## Abstract

Land surface temperature over the past decades has shown a faster warming trend during the night than during the day. Extremely low night temperatures have occurred frequently due to the influence of land-sea thermal difference, topography and climate change. This asymmetric night temperature change is expected to affect plant ecophysiology and growth, as the plant carbon consumption processes could be affected more than the assimilation processes because photosynthesis in most plants occurs during the daytime whereas plant respiration occurs throughout the day. The effects of high night temperature (HNT) and low night temperature (LNT) on plant ecophysiological and growing processes and how the effects vary among different plant functional types (PFTs) have not been analyzed extensively. In this meta-analysis, we examined the effect of HNT and LNT on plant physiology and growth across different PFTs and experimental settings. Plant species were grouped according to their photosynthetic pathways (C_3_, C_4_, and CAM), growth forms (herbaceous, woody), and economic purposes (crop, non-crop). We found that HNT and LNT both had a negative effect on plant yield, but the effect of HNT on plant yield was primarily related to a reduction in biomass allocation to reproduction organs and the effect of LNT on plant yield was more related to a negative effect on total biomass. Leaf growth was stimulated at HNT and suppressed at LNT. HNT accelerated plants ecophysiological processes, including photosynthesis and dark respiration, while LNT slowed these processes. Overall, the results showed that the effects of night temperature on plant physiology and growth varied between HNT and LNT, among the response variables and PFTs, and depended on the magnitude of temperature change and experimental design. These findings suggest complexities and challenges in seeking general patterns of terrestrial plant growth in HNT and LNT. The PFT specific responses of plants are critical for obtaining credible predictions of the changes in crop production, plant community structure, vegetation dynamics, biodiversity, and ecosystem functioning of terrestrial biomes when asymmetric night temperature change continues.

## Introduction

The increased intensity of human activities has been magnifying the climate change and its consequences in recent decades (IPCC, 2013). A remarkable feature of climate change is global warming, caused by anthropogenic emissions of key greenhouse gasses that absorb infrared radiation, such as CO_2_, CH_4_, and N_2_O, deforestation and urbanization. The global temperature is forecasted to continuously increase 1–3.7°C by the end of the 21st century (IPCC, 2013). Compared with day temperature, night temperature has increased faster at local (Peng et al., 2004), country (Zhou et al., 2004; Rao et al., 2014), and global scales (Vose et al., 2005). On average, the lowest land nighttime temperature increased about 0.2°C per decade between 1950 and 1993, which is double the increased highest daytime temperature (IPCC, 2001). It is probably due to the incremental cloudiness, which leads to less radiant heat loss (Alward et al., 1999). Night temperature increased 1.13°C in the Philippines from 1979 to 2003 (Peng et al., 2004), whereas night temperature in Lybia over a period of 45 years (1950–1995) increased at a rate of 0.18°C per decades (Jones et al., 1999). Based on the prediction of multi-model ensembles, asymmetric warming between day and night is going to continue in the future (Christensen et al., 2007; Sillmann et al., 2013). Therefore, plants in the future will be exposed to warmer nights, which could greatly influence crop yield and vegetation dynamics as well as ecosystem biodiversity, structure and productivity.

Due to the influence of land-sea thermal differences, topography and climate change, extremely low temperatures have also occurred frequently around the world (Yang et al., 2006). Low temperature is one of the major environmental factors impacting plant growth, development and ecological distribution (Allen and Ort, 2001). A variety of crops from tropical and sub-tropical regions, such as maize, tomato, cucumber, and mango, are sensitive to cold when cultivated in temperate environments (Jones and Ort, 1998; Allen and Ort, 2001; Meng et al., 2008). As people have begun introducing plants from warm climates into cool climates, it has become important to understand the effects of LNT stress, which needs substantially more research.

Studies on plant response mechanisms to warming or chilling temperatures serve a great purpose in understanding agriculture and natural ecosystems. Increased research efforts have used manipulated field experiments across the world to investigate the potential impacts of climate warming on terrestrial plants and ecosystems (Rustad, 2008). However, the majority of these previous studies have focused on the increase of daily or monthly mean temperature, assuming no difference in the impact of day versus night temperature (Peng et al., 2004). Rustad et al. (2001) conducted a meta-analysis of experimental data from ecosystem warming studies and found that elevated temperatures significantly increased above ground productivity by 19%. In fact, the effects of night temperature are different from that of day temperature (Xia et al., 2014) and produced a relatively greater challenge in estimating global change impact on crop yield and ecosystem functions (Jagadish et al., 2015). Previous studies on night temperatures have focused either on the effects of HNT and LNT alone (Friend, 1981; Seddigh and Jolliff, 1984a,b,c; Koscielniak, 1993; Bertamini et al., 2005) or the mixed effects of night temperatures and CO_2_ concentration (Mortensen and Moe, 1992; Volder et al., 2004; Cheng et al., 2008, 2009, 2010), light period (Gimenez and Rumi, 1988; Turner and Ewing, 1988; Lee et al., 1991; Verheul et al., 2007), intensity (Bunce, 1985; Mortensen, 1994; Rapacz, 1998; Flexas and Osmond, 1999; Davies et al., 2002) as well as other environmental factors (Schoppach and Sadok, 2013) and growth regulators (Shah et al., 2011; Mohammed et al., 2013; Zhang et al., 2014). These experiments had been conducted on pineapple (Neales et al., 1980), peanut (Bagnall et al., 1988; Wang, 2007; Lin et al., 2011) and shrub-grass ecosystems (Beier et al., 2004). Although the interest in the influence of night temperatures on many aspects of plants is growing, studies are scattered and there lacks a synthetic study on how and to what extent night temperature change impacts terrestrial plant growth and biomass accumulation. To accurately predict the effects of climatic change and develop sound adaptive agricultural systems and land management practices, it is imperative to understand how night temperature affects photosynthetic carbon gain, loss and allocation through a comprehensive analysis of HNT and LNT studies.

Night temperature has both direct and indirect effects on plant physiology, morphology, growth and yield. HNT and LNT impact plant physiology in many aspects, of which photosynthesis is the most severely affected process (Berry and Bjorkman, 1980; Damian and Donald, 2001; Yu et al., 2002; Liu et al., 2010, 2011). There was a consistent suppression on *A*_net_ (net CO_2_ assimilation rate) at LNT for both C_3_ (Flexas and Osmond, 1999; Bange and Milroy, 2004; Zhang et al., 2010; Sao et al., 2013b) and C_4_ species (Sao et al., 2013a), but a stimulation for CAM species (Chen et al., 2008; Pollet et al., 2011). HNT had a positive (Seddigh and Jolliff, 1984c; Prieto et al., 2009; Darnell et al., 2013), negative (Teragishi et al., 2001; Mohammed et al., 2013; Narayanan et al., 2015; Peraudeau et al., 2015), or no effect (Veatch et al., 2007; Ibrahim et al., 2010; Cheesman and Klaus, 2013) on *A*_net_ for C_3_ species and a negative (Prasad and Djanaguiraman, 2011) effect for C_4_ species. The effect of HNT and LNT on photosynthesis was related to leaf chlorophyll content (Prasad and Djanaguiraman, 2011), fluorescence parameters including photochemical efficiency of PSII (*F*_v_/*F*_m_), PSII quantum yield (Φ_PSII_) and ETR (Liu et al., 2011, 2012; Zhang et al., 2014), nitrogen (N) concentration (Mohammed and Tarpley, 2009a), *g*_s_ (stomatal conductance) (Farquhar and Sharkey, 1982) and enzyme activities related to carbon fixation (Noctor and Foyer, 1998). Among different PFTs, a positive correlation between HNT and plant height was reported (Patterson, 1990; Papadopoulos and Hao, 2000; Cheng et al., 2009; Lucidos et al., 2013). However, LNT had a negative effect on plant height for C_3_ (Zieslin et al., 1986; Pressman et al., 2006; Kjær et al., 2008) and C_4_ species (Uehara et al., 2009), but a positive effect for CAM species (Serra and Carrai, 1988). The responses of biomass accumulation to different night temperature conditions were not identical among different species. HNT had a positive effect on stem dry weight for woody plants (Malek et al., 1992; Cheesman and Klaus, 2013), a positive (Cheng et al., 2008, 2009; Darnell et al., 2013) or negative (Seddigh and Jolliff, 1984a; Lee and Myeongwhan, 2011) effect for herbaceous plants. However, LNT had a positive (Lepage et al., 1984), negative (Kjær et al., 2008; Uehara et al., 2009; Rehmani et al., 2014) or no effect (Dejong and Smeets, 1982) for herbaceous plants. Both HNT and LNT resulted in a reduction in crop yield, such as rice (Ziska and Manalo, 1996; Kanno and Makino, 2010; Mohammed and Tarpley, 2010; Shi et al., 2013), winter wheat (Zhang Y. H. et al., 2013; Narayanan et al., 2015), sorghum (Prasad and Djanaguiraman, 2011) and tomato (Khayat et al., 1985; Zhang et al., 2010; Qi et al., 2011; Zhang Y. et al., 2013). Clearly, lessons from previous studies are not all consistent and sometimes, contradictory. It is essential to conduct a comprehensive review on the responses of different plant functional groups to different night temperatures.

In addition to species functional groups and night temperature treatments, experimental design (e.g., treatment duration and growth facility) may also matter in understanding plant responses to night temperature change. A significant reduction in rice yield at HNT was associated with the reduction of N and non-structural content translocation after flowering in a field experiment (Shi et al., 2013). In a pot-growing experiment, yield loss was concerned with decreased dry matter allocation to grain due to reduced spikelet fertility during the reproductive stage (Cheng et al., 2009). The variation of *A*_net_ deduction due to HNT and LNT was dependent on experimental durations (Teragishi et al., 2001; Zhu et al., 2005; Ibrahim et al., 2010; Sao et al., 2010; Prasad and Djanaguiraman, 2011). However, the difference between responses to different treatment durations or to growing facilities is ambiguous. Confirming the effects of experimental methodology is of key theoretical and practical significance to help agriculture to choose the right cultivation practice to mitigate adverse effects caused by HNT or LNT.

A meta-analysis of plant responses to temperature indicated that CO_2_ elevation affected plant ecophysiology and growth, with different magnitudes at different temperature treatments (Wang et al., 2012). Not only daily temperature, but also the magnitude of night temperature variation caused different impacts. Elevated night temperature by 5°C had a negative effect on *A*_net_ and *g*_s_ but no effect on intercellular CO_2_ concentration (*C*_i_) (Mohammed et al., 2013), while elevated night temperature by 8°C significantly increased *A*_net_, *g*_s_, and *C*_i_ of bell peppers (Darnell et al., 2013). With more night temperature reduction, the decrease in total dry weight, number of leaves and leaf area for goatsrue were increased gradually (Patterson, 1993). Although the effects of different magnitude of night temperature variation on plant physiology and growth varied significantly, the comparisons between these effects are missing and a quantitative review would reveal the optimal night temperature for different ecophysiological processes and growth.

The primary objective of this study was to investigate the effects of high and LNTs on various aspects of plant responses, including physiological, morphological, and growth characteristics. Specifically, our objectives were to: (1) assess the difference and magnitude of HNT and LNT effects on plant physiology, morphology and yield-related responses. The physiological characters included *A*_net_, PSII function, *g*_s_, dark respiration (*R*_d_), maximum carboxylation rate (*V*_cmax_), maximum ETR (*J*_max_), tissue N and TNC. The morphology responses included plant height and leaf characteristics (number of leaves, LAI, SLA and LAR). Yield-related parameters included dry biomass, number of reproductive organs and yield; (2) detect differences among different PFTs based on photosynthetic pathways, growth forms and economic value; (3) investigate the effect of the magnitude of the night temperature changes on different responses; (4) tease apart the effect of growth facilities or treatment durations on affecting these responses. Accordingly, the specific hypotheses were proposed as: (1) HNT and LNT would have similar negative effects on plant physiological performance and growth; (2) LNT would have a stronger negative effect on C_4_ species than on C_3_ species; HNT would have a stronger negative effect on C_3_ than on C_4_ species. To test these hypotheses, we conducted a comprehensive meta-analysis of night-temperature manipulated studies published from 1980 through 2015, using the response ratio lnr as an estimate of the effect size of night-temperature relative to control plots.

## Materials and Methods

### Data Selection

Peer-reviewed journal publications were searched with the key word “night temperature” on the Web of Science to build a comprehensive database. The list of papers were then cross-checked with a list of references cited in review articles that were relevant to night temperature effects in order to assure that all articles available for this meta-analysis were included. Any article published in English from 1980 to 2015 that met the following two criteria were included: (1) plants were treated at ANT as a control group, and HNT or LNT as treatment groups; (2) measurements on physiology, morphology, and yield were carried out on both control and treatment groups. The following two criteria were applied to exclude studies: (1) day and night temperatures were treated at the same time; (2) studies focused on extreme temperature values, which resulted in the death of plants. Eventually, 112 papers were selected in this study (Supplementary Material S1). Data were extracted directly from the tables in the articles or were obtained by using the software GetData Graph Digitizer when presented in graphical formats. In these studies, night temperature was 1–20°C above or below ANT, with only four studies more than 20°C above or below ANT (Supplementary Figure S2). Response variables extracted from these articles contained physiological characters including net photosynthesis (*A*_net_), PSII efficiency (*F*_v_/*F*_m_), stomatal conductance (*g*_s_), dark respiration (*R*_d_), non-structural carbohydrate content (TNC), tissue nitrogen (N) content (i.e., stem, leaf, panicle, spike, grain, shoot, root, and total N) and tissue carbon (C) content (i.e., stem, leaf, shoot, root, and total C), morphological features (i.e., plant height, stem diameter, internode length, number of leaves, SLA, LAI, LAR) and yield-related parameters (i.e., dry weight, number of reproductive organs, days to flowering and yield). For multi-year studies on annual species, results from different seasons were considered independent and all observations were included in this analysis. To ensure the independent nature of the data, we excluded duplicate results found in different publications. However, our analyses were not completely independent because individual papers often provided data with more than one treatment (e.g., different HNT or LNT magnitudes) and/or different response variables. To examine the influence of non-independence of data, we first averaged those data from the same published study by PFTs so that only one comparison was used from a published study for each PFT. Nonetheless, we found that most of the response patterns were unchanged; therefore, all data were used in our study.

### Categorization of the Studies

Night temperature was categorized into three levels: ANT, HNT, and LNT. In addition to the response variables and night temperature categories described above, plant species, sample sizes, growth facilities and treatment durations under each temperature treatment were also collected. Following the categorization of Wang et al. (2012), plant species were classified based on photosynthetic pathways (C_3_, C_4_, or CAM), growth forms (herbaceous or woody) and economic values (crop or non-crop). We listed the species, PFTs and associated references used in this study (Supplementary Table S3). The experiments conducted in these studies were either indoors (growth chamber and greenhouse) or field studies. Due to relatively less data in the field studies, growth facilities used in these experiments were categorized as two levels of pot size: <10L and >10L. Because the treatment duration varied from hours to years, we grouped then into two levels: short-term (hours–days) and long-term (months–years).

### Meta-Analysis Methods

We employed a similar method from Hedges et al. (1999). To avoid the adverse effects of different units, we used the response ratio *r* = *X*_t_/*X*_c_ to estimate the effect size of night temperature treatments, where *X*_t_ is the treatment mean and *X*_c_ is the control mean. In order to compare expediently, we calculated the natural logarithm of the response ratio (lnr). In addition to the mean value, standard deviation (SD) and sample size (*n*) for each individual observation were also collected to calculate the variance of effect size. Using METAWIN software 2.1 (Sinauer Associates, Inc. Sunderland, MA, USA), we calculated the effect size of the targeted variables and used a weighted, fixed-effects model to evaluate the categorical effects on night temperature treatments, plant species, pot sizes and treatment durations. If the 95% confidence interval (CI) of lnr generated by the fixed-effects model overlapped 0, the temperature treatment was considered to have no significant impact on the response variables. If the upper bound of the 95% CI was smaller than 0, the response was considered significantly negative. Conversely, it indicated that the treatment had a significantly positive effect on variables if the lower bound of the 95% CI was greater than 0. Although total difference among groups was divided into within-group and between group difference, the significance level of the latter revealed whether the response was different among groups (Hedges et al., 1999). The response of plants was considered significantly disparate between HNT and LNT overall or for different species, pot size or treatment duration if their 95% CIs did not overlap. Significance was established at *p* < 0.05 unless otherwise noted.

Publication bias of the effect size (lnr) in this meta-analysis was determined with METAWIN software 2.1 (Sinauer Associates, Inc. Sunderland, MA, USA). We calculated Spearman’s rank-order correlation (*r*_s_) which indicates the relationship between the effect size (lnr) and the sample size (Begg and Mazumdar, 1994), and Rosenthal’s fail-safe number which represents the number of additional studies with a mean effect size of zero needed to eliminate the significance of a significant effect (Rosenthal, 1979). Publication bias was significant if *p*-value of *r*_s_ was smaller than 0.05. However, the publication bias may be safely ignored if the fail-safe number is larger than a critical value of 5n+10 where *n* is the number of studies (Rosenberg, 2005).

### Statistical Analysis

Original data collected from these studies were arranged into a database in which the value of response variables was lnr. The relationship between lnr of all the variables and the magnitude of night temperature treatments were evaluated by a second-degree polynomial or linear regression analysis with the R statistical programming language (R 3.2.2 for Windows GUI front-end).

## Results

### Significance of HNT and LNT

**FIGURE 1:**
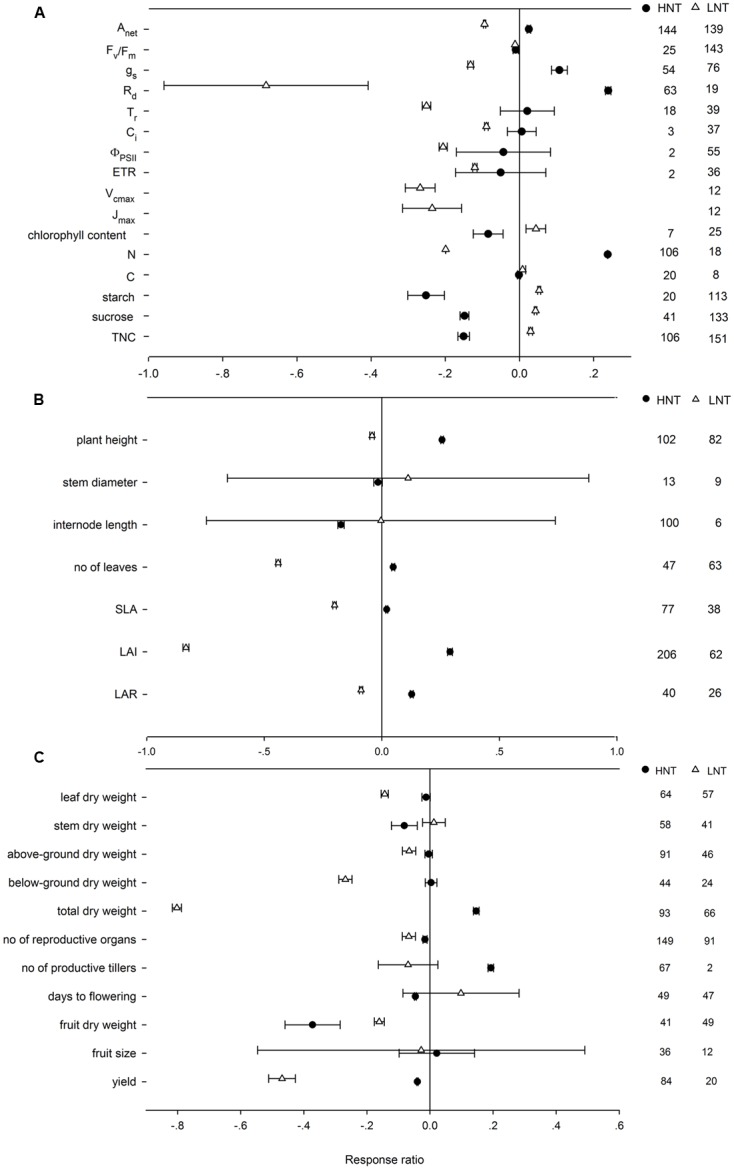
**Plant (A)** physiological, **(B)** morphological, and **(C)** yield-related responses to HNT (filled circles) and LNT (open triangle). Each data point represents the mean ± 95% confidence interval (CI). The number of observations for each variable is given on the right of the graph. Note that N is tissue nitrogen content including stem, leaf, panicle, spike, grain, shoot, root, and total N. C is tissue carbon content including stem, leaf, shoot, root, and total C.

Across all of the studies, HNT increased *A*_net_, *g*_s_, *R*_d_, and tissue *N* content on average by 2.56, 11.37, 27.02, and 26.87%, respectively, decreased *F*_v_/*F*m, chlorophyll content, starch, sucrose and TNC content by 0.98, 8.08, 22.26, 13.77, and 13.97%, but unaffected *T*_r_ (transpiration rate), *C*_i_, PSII quantum yield, ETR and tissue C content (**Figure 1A**). LNT had negative effects on most physiological response variables by different magnitudes, but increased chlorophyll (4.81%), C (1.11%), starch (5.73%), sucrose (4.71%) and TNC content (3.32%). HNT decreased stem diameter and internode length by 1.61%, and 15.97%, which were unchanged by LNT (**Figure 1B**). HNT and LNT had an opposite effect on plant height, number of leaves, SLA, LAI, and LAR (**Figure 1B**). HNT had positive effects on total dry weight and number of productive tillers, negative effects on leaf, stem, and fruit dry weight, number of reproductive organs, flowering time and yield, and no effects on above-ground, below-ground dry weight and fruit size (**Figure 1C**). LNT decreased leaf (13.69%), fruit (15.18%), above-ground (6.7%), below-ground (23.8%), and total dry weight (55.33%), reproductive organs number (6.82%) and yield (37.66%), respectively, but had no effects on stem dry weight, number of productive tillers, anthesis and fruit size (**Figure 1C**). Among all the variables, there was publication bias for chlorophyll content (*r*_s_ = -0.399, *p* = 0.02), leaf (*r*_s_ = 0.346, *p* = 0.002), stem (*r*_s_ = 0.339, *p* = 0.0006), above-ground (*r*_s_ = 0.235, *p* = 0.006), and below-ground dry weight (*r*_s_ = 0.22, *p* = 0.07), which could not be ignored based on Rosenthal’s value.

### Variable Responses among Plant Functional Types (PFTs)

**FIGURE 2:**
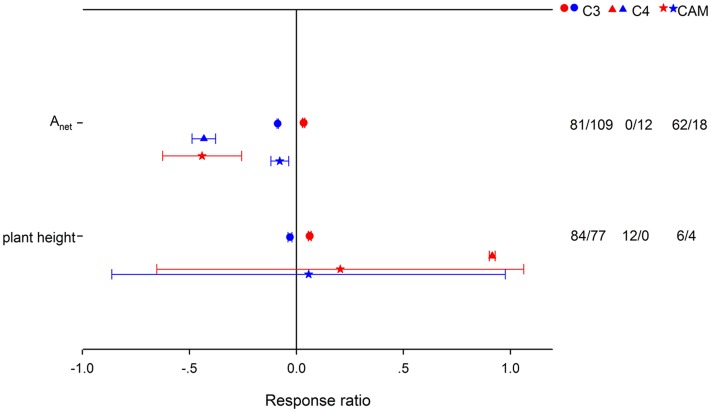
**Photosynthetic rate (*A*_net_) and plant height responses to HNT (red) and LNT (blue) in C_3_ (circles), C_4_ (triangles), and CAM (stars) species.** Each data point represents the mean ± 95% CI. The number of observations for each variable is given on the right of the graph.

**FIGURE 3:**
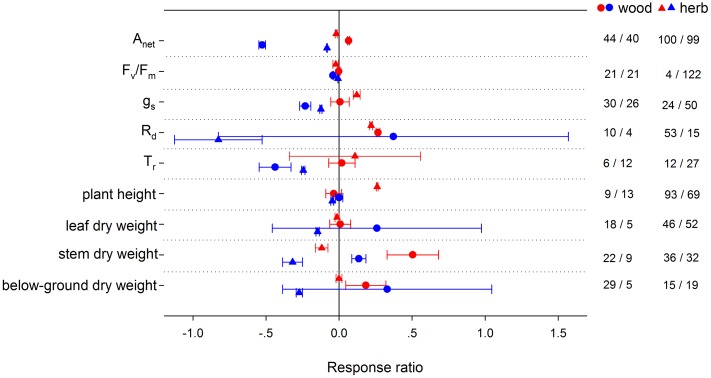
**Plant physiological, morphological and yield-related responses to HNT (red) and LNT (blue) in woody (circles) and herbaceous (triangles) species.** Each data point represents the mean ± 95% CI. The number of observations for each variable is given on the right of the graph.

HNT stimulated *A*_net_ by 3.43% for C_3_ species, but suppressed it by 35.57% for CAM species (**Figure 2**). Note that there were not enough publications for a summary on C4 species. LNT suppressed *A*_net_ more for C_4_ species than for C_3_ and CAM species. HNT increased plant height differently for C_3_ and C_4_ species by 6.41 and 150%, respectively. For woody species, *A*_net_, *R*_d_ and biomass (stem and below-ground) responded more positively, while *g*_s_ and plant height responded less positively to HNT than for herbaceous species (**Figure 3**). The LNT effect on woody and herbaceous species was significant for *A*_net_, *g*_s_, *T*_r_, stem dry weight and plant height. LNT had a less negative effect on *A*_net_, *T*_r_, and *g*_s_ but a larger negative effect on stem dry weight and plant height in herbaceous species than in woody species.

**FIGURE 4:**
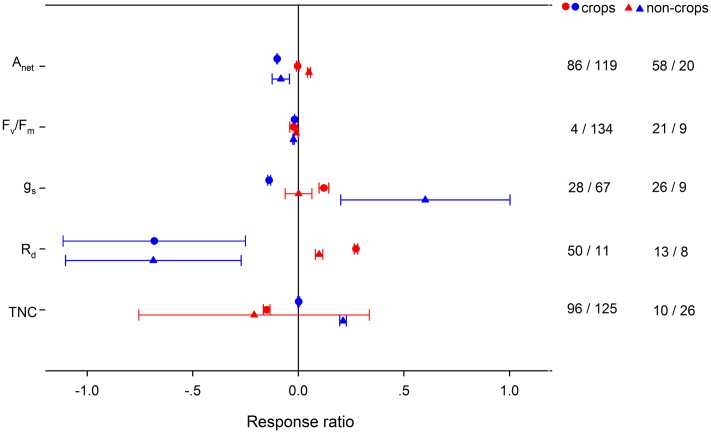
**Plant physiological responses to HNT (red) and LNT (blue) in crops (circles) and non-crops (triangles) species.** Each data point represents the mean ± 95% CI. The number of observations for each variable is given on the right of the graph.

**FIGURE 5:**
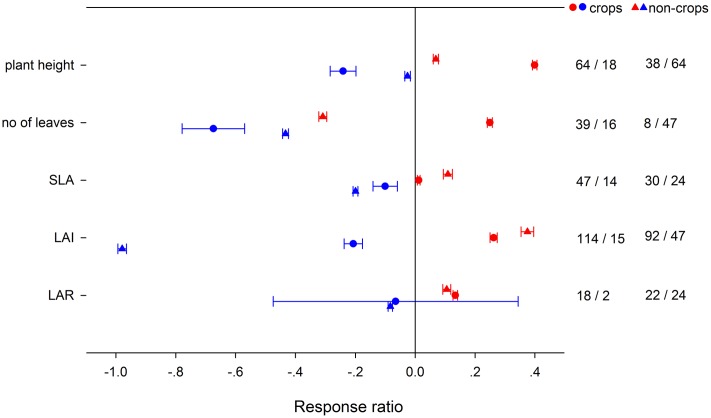
**Plant morphological responses to HNT (red) and LNT (blue) in crops (circles) and non-crops (triangles) species.** Each data point represents the mean ± 95% CI. The number of observations for each variable is given on the right of the graph.

**FIGURE 6:**
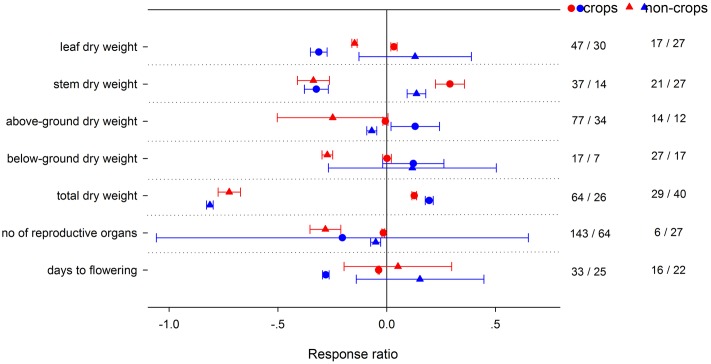
**Plant yield-related responses to HNT (red) and LNT (blue) in crops (circles) and non-crops (triangles) species.** Each data point represents the mean ± 95% CI. The number of observations for each variable is given on the right of the graph.

High night temperature had a greater positive effect for *g*_s_ and *R*_d_ in crops and *A*_net_ in non-crops (**Figure 4**). Positive effects of LNT on *g*_s_ and TNC were significantly greater for non-crops than for crops. Positive effects of HNT on plant height, number of leaves and LAR were greater in crops, but the effects on SLA and LAI were greater in non-crops (**Figure 5**). LNT decreased plant height and number of leaves more in crops, but decreased SLA and LAI more in non-crops. HNT had positive effects on leaf, stem and total dry weight for crops but negative effects on non-crops (**Figure 6**). For below ground, dry weight and number of reproductive organs, non-crops responded more negatively to HNT than crops. LNT had positive effects on above-ground and total dry weight for crops and negative effects for non-crops, while stem dry weight in crops and non-crops responded to LNT oppositely. LNT had a greater negative effect on leaf dry weight and anthesis for crops than for non-crops.

### Magnitude-Introduced Uncertainty

**FIGURE 7:**
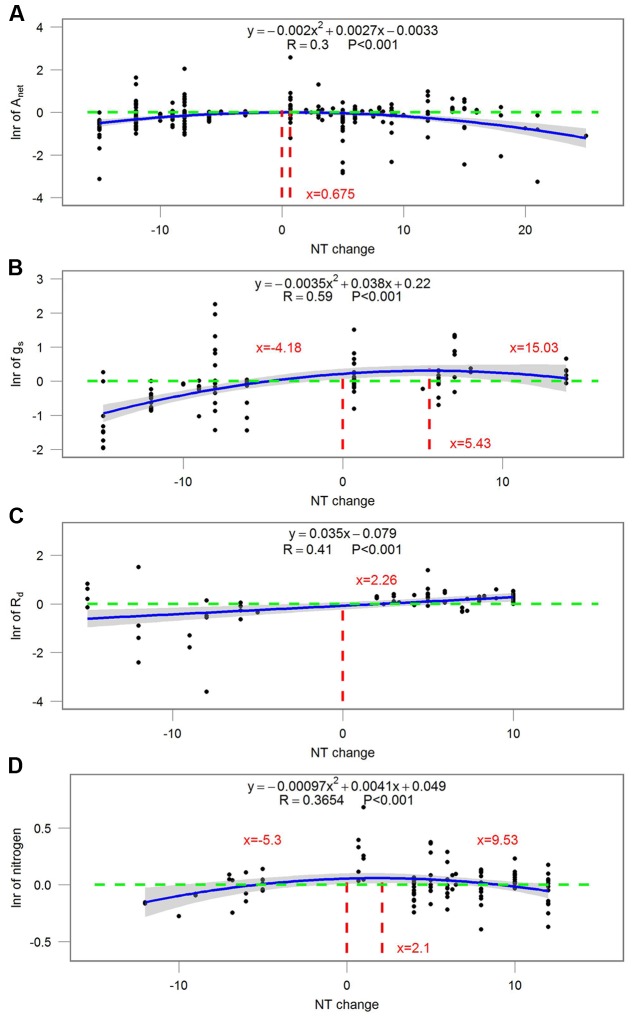
**Correlations between the magnitude of NT treatment and the response ratio of (A)** net photosynthetic rate (*A*_net_); **(B)** stomatal conductance (*g*_s_); **(C)** dark respiration rate (*R*_d_) and **(D)** tissue nitrogen content (N). Each point represents response ratio (lnr) to HNT or LNT. Regression function, variation coefficient and *p*-value are presented in the middle of each graph. Different lines indicate *X* = 0 (red line), *x*-value when *y* is the maximum, crossing points of *y* = 0 (green line) and regression relationships. Note that N is tissue nitrogen content including stem, leaf, panicle, spike, grain, shoot, root, and total N.

**FIGURE 8:**
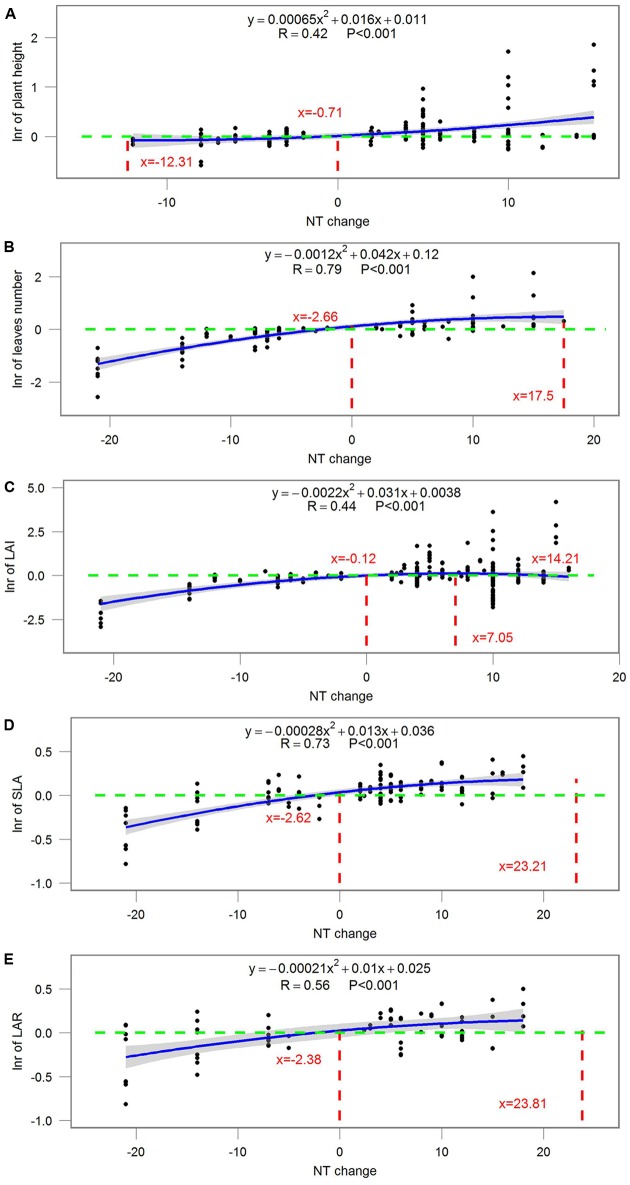
**Correlations between the magnitude of NT treatment and the response ratio of (A)** plant height; **(B)** number of leaves; **(C)** leaf area index (LAI); **(D)** specific leaf area (SLA); **(E)** leaf area ratio (LAR). Each point represents response ratio (lnr) to HNT or LNT. Regression function, variation coefficient and *p*-value are presented in the middle of each graph. Different lines indicate *X* = 0 (red line), *x*-value when *y* is the maximum, crossing points of *y* = 0 (green line) and regression relationships.

**FIGURE 9:**
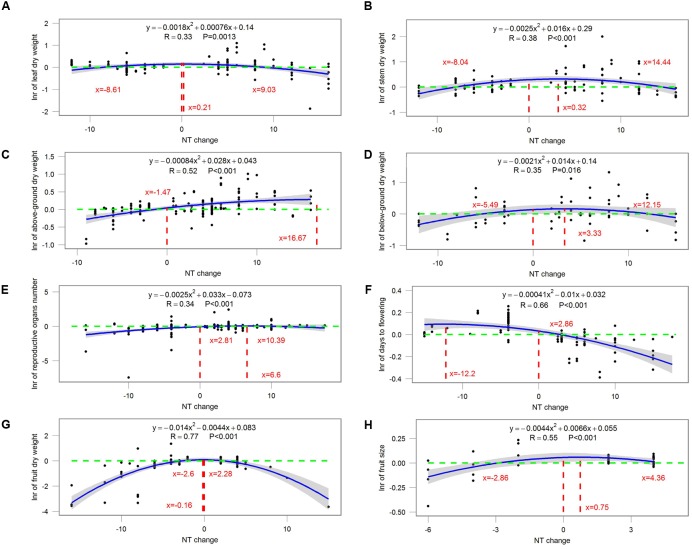
**Correlations between the magnitude of NT treatment and the response ratio of (A)** leaf dry weight; **(B)** stem dry weight; **(C)** above-ground dry weight; **(D)** below-ground dry weight; **(E)** number of reproductive organs; **(F)** days to flowering; **(G)** fruit dry weight and **(H)** fruit size. Each point represents response ratio (lnr) to HNT or LNT. Regression function, variation coefficient and *p*-value are presented in the middle of each graph. Different lines indicate *X* = 0 (red line), *x*-value when *y* is the maximum, crossing points of *y* = 0 (green line) and regression relationships.

Most ecophysiological and growth parameters formed a quadratic relationship, except for *R*_d_, which responded linearly, to night temperature treatment (**Figures 7–9**). *A*_net_, *g*_s_, and tissue *N* were the highest when NT was 0.675, 5.43, and 2.1°C above ambient temperature, respectively (**Figure 7**). Morphological parameters, including number of leaves, LAI, SLA, and LAR, formed downward-opening parabola relationships with night temperature change, while plant height, on the other hand, formed an upward-opening parabola relationship with night temperature change (**Figure 8**). Yield-related parameters including leaf, stem, above-ground and below-ground dry biomass as well as the number of reproductive organs, days to flowering, fruit size and fruit weight had downward-opening quadratic relationships with night temperature change (**Figure 9**).

### Uncertainty Related to the Experimental Design

**FIGURE 10:**
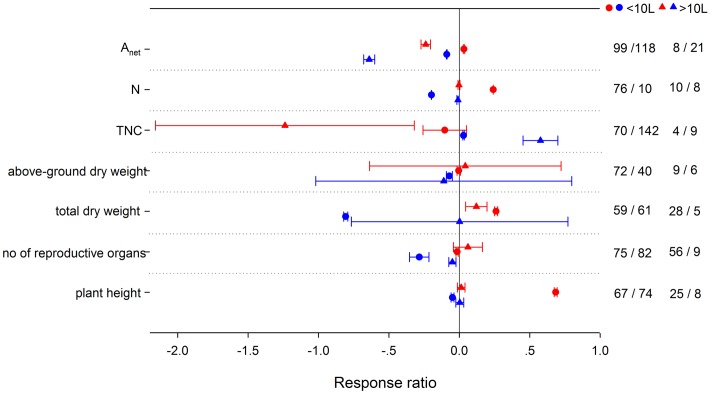
**Plant physiological, morphological and yield-related responses to HNT (red) and LNT (blue) grown in <10 L pots (circles) or >10L pots (triangles).** Each data point represents the mean ± 95% CI. The number of observations for each variable is given on the right of the graph. Note that N is tissue nitrogen content including stem, leaf, panicle, spike, grain, shoot, root, and total N.

Pot size involved in the experiments was a significant factor influencing the effects on *A*_net_, tissue N, TNC, total dry weight, number of reproductive organs, and plant height’s responses to HNT (**Figure 10**). Plants in smaller pots (<10L) responded more positively for *A*_net_, *N*, *w*_t_, and plant height to HNT. TNC, however, responded more negatively at HNT in larger pots. *N*, *w*_t_, number of reproductive organs and plant height were reduced more in smaller pots at LNT. *A*_net_ was decreased more and TNC was increased more in larger pots with LNT.

**FIGURE 11:**
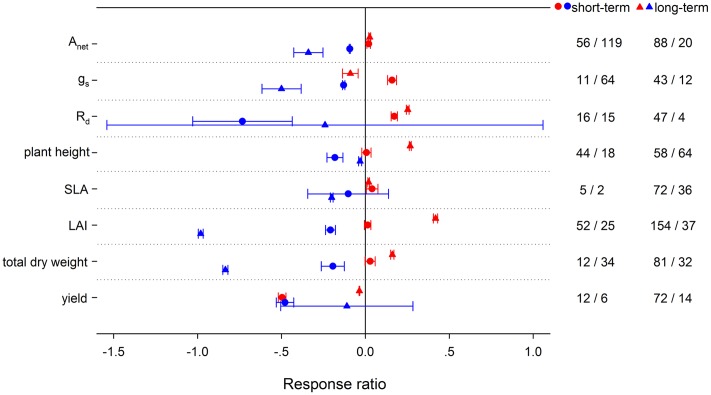
**Plant physiological, morphological and yield-related responses to HNT (red) and LNT (blue) for different treatment durations (circles, short-term; triangles, long-term).** Each data point represents the mean ± 95% CI. The number of observations for each variable is given on the right of the graph.

Experimental duration also played an important role in affecting plant responses to HNT and LNT. HNT increased respiration less in short-term treatments than that in long-term treatments, while LNT duration had insignificant effects (**Figure 11**). Stomatal conductance was significantly increased at short-term HNT but decreased at long-term HNT. Compared with short-term duration, long-term LNT caused more reduction on *A*_net_ and *g*_s_. Experimental duration had no effects on the responses of SLA to HNT or to LNT, but generated different effects on plant height and LAI at both HNT and LNT. Long-term HNT increased plant height and LAI more, whereas short-term LNT reduced plant height more and LAI less. For total dry weight, long-term HNT and LNT had greater effects than short-term. Yield was decreased more at short-term HNT and different durations had no significant effects in affecting yield responses to LNT.

## Discussion

Asymmetric night warming and chilling have long been acknowledged as a universal phenomenon in recent years and caused great agricultural, economic and ecological consequences (Xia et al., 2014). At the leaf and organism level, however, comprehensive quantitative analysis of the effect of HNT and LNT on plants ecophysiology and growth remain unclear. In this study, we collected data from night temperature manipulative studies and analyzed the changes in ecophysiological and whole-plant responses due to changes in night temperatures. Overall, we found that: (1) the significance and degree of the effect of HNT and LNT and the causes of yield reduction at HNT and LNT were different; (2) there existed significant variations among different PFTs in responding HNT and LNT; (3) there was an optimal night temperature for important processes of plants physiology and growth; (4) the responses to HNT and LNT appeared dependent of the experimental designs.

### Plant Responses to HNT and LNT

Consistent with our hypothesis, both HNT and LNT had a negative effect on plants yield, with a greater negative effect at LNT, probably due to a greater night temperature reduction for LNT treatments (Supplementary Figure S2). HNT and LNT are considered great threats to plants production, especially for crops (Hall, 2000; Maali and Heidarvand, 2010). The impacts of temperature change on plant yield have been assessed directly through greenhouse (Cheng et al., 2009; Mohammed and Tarpley, 2009a,b; Kanno and Makino, 2010; Zhang et al., 2010; Qi et al., 2011) and field experiments (Peng et al., 2004; Nagarajan et al., 2010; Shi et al., 2013; Zhang Y. H. et al., 2013). The long-term effect of climate change on crops was also estimated through crop-growth models, such as CERES (Ritchie and Otter, 1985; Jones et al., 1986), ORYZA2000 (Bouman and Laar, 2006), and CropSyst (Stockle et al., 1994). Warming stress triggered a significant loss of crop yield worldwide, particularly in nations such as China (Li et al., 2004), Japan (Hasegawa et al., 2009), Philippines (Peng et al., 2004), as well as nations in south and southeast Asia (Welch et al., 2010). The deduction of yield was often associated with the decrease in the number of panicles (Peng et al., 2004), grain maturity (Suzuki and Moroyu, 1962; Ziska and Manalo, 1996) and final grain weight (Morita, 2005; Kanno and Makino, 2010), spikelet number per plant (Morita et al., 2002, 2004; Peng et al., 2004) and spikelet fertility (Cheng et al., 2009; Mohammed and Tarpley, 2009a, 2010), accelerative respiration rates (Mohammed and Tarpley, 2010), grave membrane leakage (Mohammed and Tarpley, 2009b), lower pollen germination (Mohammed and Tarpley, 2009a), and poor assimilates and *N* translocation to grains (Morita, 2005; Cheng et al., 2009; Kanno and Makino, 2010; Shi et al., 2013).

We found that the negative effect of HNT on yield was associated with a reduction in number of reproductive organs, fruit dry weight, and time for flowering. The reproductive process was regarded as most susceptible to heat stress (Prasad et al., 2006; Jagadish et al., 2007, 2008, 2010), with limited pollen viability as the major cause of yield reduction (Yoshida et al., 1981; Ziska and Manalo, 1996; Jagadish et al., 2010; Zhang et al., 2010; Zhang Y. H. et al., 2013; Fang et al., 2013; Rehmani et al., 2014). Decreased seed yield and lower seed-set under HNT were also reported in wheat (Prasad et al., 2008), rice (Mohammed and Tarpley, 2009b), cowpea (Ahmed et al., 1993), and tomato (Peet and Bartholemew, 1996). Low temperature is one of the most important abiotic stresses for plant growth, development, energy distribution (Xiong et al., 2002; Oufir et al., 2008), and yield (Kasuga et al., 1999; Lang et al., 2005). The negative effect of LNT on yield in this study appeared to be associated with a decline in the number of reproductive organs, fruit dry weight, as well as above-ground, below-ground, and total dry weight. Clearly, the mechanisms for the negative effect of HNT and LNT on plant yield were different (**Figure 1**). The negative effect of LNT on plant yield was primarily related to the negative effect of LNT on the total biomass accumulation, but the negative effect of HNT on plant yield was caused mostly by the reduced allocation of biomass to reproductive organs, as the total biomass was even stimulated by HNT. HNT had been shown to cause no change, or indeed an increase, in total biomass accumulation in crop plants such as rice (Ziska and Manalo, 1996; Cheng et al., 2009), sorghum and sunflower (Manunta and Kirkham, 1996), tobacco (Camus and Went, 1952), and cotton (Königer and Winter, 1993).

The balance between photosynthesis and respiration controls plant growth. Several recent meta-analyses of plant responses to increasing temperature had highlighted how plants may be particularly vulnerable to increases in both HNT and LNT (Lin et al., 2010; Way and Oren, 2010). In contrast to the prediction that *A*_net_ is constrained at both LNT and HNT (Berry and Bjorkman, 1980), HNT increased *A*_net_ by 2.56% and LNT decreased *A*_net_ by 8.73% among the plants included in this study (**Figure 1A**). The positive effect of HNT on *A*_net_ could be associated with the increment of *g*_s_ and tissue N, instead of PSII quantum yield and ETR (**Figure 1A**). HNT increased g_s_ of two rice genotypes (Shah et al., 2011) and wheat (Lu et al., 1998). HNT enhanced nitrogen soil mineralization (Sardans et al., 2008; Patil et al., 2010) and therefore increased leaf N concentrations (Rustad et al., 2001), which was closely related to photosynthetic capacity (Mae, 1997; Llorens et al., 2003). The loss of chlorophyll owing to HNT has been reported in many crops (Reynolds et al., 1994; Guo et al., 2006). The negative effect of LNT on *A*_net_ was associated with the negative effect on *g*_s_, PSII function (Φ_PSII_, ETR, and *F*_v_/*F*_m_), ribulose-1,5-bisphosphate carboxylase (RuBP) inactivation and leaf N (**Figure 1A**), consistent with other studies (Allen and Ort, 2001; Huang and Guo, 2005; Bertamini et al., 2007). Photosystem II has been regarded as the most sensitive to LNT (Huang et al., 2010), as LNT induced PSII photoinhibition and caused reversible inhibition of photosynthetic capacity (Dongsansuk et al., 2013; Zhang et al., 2014).

Our meta-analysis indicated that dark respiration (*R*_d_) was increased with HNT but was decreased with LNT. Increased dark respiration in HNT was reported in rice (Mohammed and Tarpley, 2009b) and *Stipa krylovii* Roshev (Chi et al., 2013a,b) at leaf scale and in rice (Cheng et al., 2009), lettuce, tomato, soybean (Frantz et al., 2004), and cotton (Loka and Oosterhuis, 2010) at organism scale. Different from the hypothesis proposed by Peng et al. (2004) that HNT increased biomass loss by enhancing respiration, our study concluded that HNT increased total biomass but LNT decreased the total, above-ground and below-ground biomass. Total biomass was stimulated by HNT in rice (Cheng et al., 2008, 2009), *Ficus insipida* and *Ochroma pyramidale* (Cheesman and Klaus, 2013), *Rosebay rhododendron* (Starrett et al., 1993), panicum (Patterson, 1990) and soybean (Hewitt et al., 1985). Studies in natural systems have seen impacts on plant phenological development. For example, the large herbaceous perennial, *Phytolacca americana* (Phytolaccaceae) demonstrated no difference in biomass accumulation but had flower set faster as a result of HNT (Wolfe-Bellin et al., 2006). Studies in temperate North America demonstrated that HNT in urban environments increased growth rates in seedlings of *Quercus rubra* (Searle et al., 2012). Similarly, changes in *R*_d_ of non-photosynthetic tissues (Saveyn et al., 2008), or a change in carbon-use efficiency (Hansen et al., 2009), could contribute to increased growth rate under HNT.

The balance between photosynthesis and respiration also controls carbohydrate accumulation, which is essential for plant growth and development (Azcón-Bieto and Osmond, 1983; Guy et al., 1992). The processes of photosynthesis and respiration responded independently to temperature and are linked by leaf carbon status (Turnbull et al., 2002). TNCs including starch and sucrose responded differently to HNT and LNT. Although HNT stimulated both photosynthesis and respiration, carbohydrate content was significantly decreased, probably due to the imbalance of the stimulation of HNT on *R*_d_ and *A*_net_. Leaf carbohydrates synthesized during the daytime were observed to be consumed more quickly during warmer nights because of enhanced leaf respiration, which depletes foliar carbohydrates and may produce a rebound effect of compensatory stimulated photosynthesis during the following day. Evidence supporting this hypothesis has been reported in both greenhouse and field experiments (Beier et al., 2004; Lin et al., 2010). Turnbull et al. (2002) found that leaf starch concentration, soluble sugar and total non-structural carbohydrate declined significantly with the increase of nocturnal temperature. However, no compensatory effect was found between respiration and photosynthesis under nocturnal warming in Mediterranean grassland (Xia et al., 2014). Further investigation is required to discover whether the compensatory effect between respiration and photosynthesis under night warming is related to other environmental conditions, such as water and nitrogen availability. LNT increased carbohydrate content via a lesser negative effect on *R*_d_ than on *A*_net_, which was approved in many studies (Siminovitch and Briggs, 1953; Steponkus and Lanphear, 1968; Guy et al., 1980; Kaurin et al., 1981; Miao et al., 2009). The increased carbohydrate content posed a negative effect on the day-time photosynthesis at LNT.

Plants adapt to climate stresses via multiple strategies, such as the adjustments of phenology and morphology (Wahid et al., 2007). Leaf morphology was particularly sensitive to HNT as leaf expansion often reached its peak during the night (Schurr et al., 2000; Pantin et al., 2011). HNT had positive effects on SLA, LAR, number of leaves and LAI. When exposed to HNT, the expansion of leaf area and plant height benefited capturing more light for photosynthesis (Seddigh and Jolliff, 1984c; Darnell et al., 2013). Elevated water temperature, in addition to air temperature, can also stimulate leaf expansion and elongation (Tsunoda, 1964; Cutler et al., 1980). Kanno et al. (2009) attributed the positive effect of HNT on plant biomass to an increase in leaf area in rice plants, which was found in tomatoes and *Galega officinalis* (Hussey, 1965; Patterson, 1993) as well. LNT, on the other hand, suppressed plant height, number of leaves, LAI, SLA, and LAR, corresponding to the previous studies (Cockshull, 1979; Dejong and Smeets, 1982; Patterson, 1993; Kjær et al., 2007, 2008; Kjaer et al., 2010).

### Variable Responses among Plant Functional Types (PFTs)

Previous studies indicated that increased daytime temperature had stronger effects on *A*_net_ of C_3_ species than C_4_ species (Wahid et al., 2007). No consistent results for HNT effect on *A*_net_ were found due to insufficient data for C_4_ species. However, we did find that LNT decreased *A*_net_ more in C_4_ species than in C_3_ species, which was closely associated with relatively more reduction of *g*_s_, ETR, *V*_cmax_, and *J*_max_ for C_4_ species (**Figure 2**; Supplementary Figure S4). Low temperature effects on C_4_ photosynthesis have been frequently examined (Long, 1998). C_4_ photosynthesis has been suggested to be inherently sensitive to chilling because of the cold lability of the C_4_ cycle enzymes (Long, 1983; Sage, 2002; Sage and Kubien, 2007).

High night temperature stimulated *A*_net_ in woody plants but suppressed it in herbaceous plants. The same pattern was found in stem biomass. Though studies have reported a greater warming-induced stimulation in woody biomass than in herbaceous biomass (Lin et al., 2010), the result that woody plants were more favored under night warming than herbaceous plants has not been reported. The stimulation of HNT on stem biomass is greater than that on below-ground biomass for woody species, however, for herbaceous species, HNT had no effect on leaf- or below-ground biomass, yet suppressed stem biomass significantly. Our results imply that more resources will be allocated to aboveground growth, and therefore above-ground competition for resources, such as light, will be more important for woody species under night warming (Suding et al., 2005; Lin et al., 2010). In ecosystems where herbaceous and woody plants coexist, a greater biomass stimulation of woody than of herbaceous species may increasingly suppress growth, especially above-ground growth, of herbaceous species via a shading effect (Castro and Freitas, 2009). LNT also had a positive effect on woody stem biomass, which might be related to a stronger suppression in *R*_d_ than *A*_net_. The negative effect of HNT on *A*_net_ for herbaceous plant was caused primarily by damage to PSII efficiency (*F*_v_/*F*_m_) (**Figure 3**). LNT decreased *A*_net_ more in woody plants than in herbaceous plants, along with a greater decrease of *F*_v_/*F*_m_ and *g*_s_ in woody plants, which was consistent with previous studies (Sao et al., 2010, 2013b; Liu et al., 2011). It is important, however, to note that more research is needed on the effect of night temperature on plants biomass allocation, since the publication bias on this effect could not be ignored in this meta-analysis. Low sample sizes for some functional groups used in this analysis (e.g., C_4_ and woody species) require that some results in this analysis be interpreted with caution. Nevertheless, results from these under-represented groups demonstrate that further study of these groups is critical for this untested hypothesis in the future.

Teasing apart the variations of the responses among crops and non-crops is important to guide future research in agricultural practice and genetic engineering. HNT had a positive effect on *A*_net_ for non-crops but had no effect for crops, while HNT had a positive effect on *R*_d_ for both crops and non-crops. Due to different responses of *A*_net_ and *R*_d_ to HNT between crops and non-crops, TNC was decreased in crops but unchanged in non-crops. Accordingly, HNT stimulated leaf-, stem-, total dry biomass for crops but decreased leaf-, stem-, below-ground, total dry biomass for non-crops. Similarly, crops better adapted to LNT than non-crops, as LNT had a positive effect on total dry biomass for crops but negative for non-crops. The fact that crops coped with HNT and LNT better than non-crops could imply an improved stress-tolerant ability for improved crops through breeding, genetic engineering, and management practices. Changes in HNT and LNT could influence vegetation dynamics and ecosystem structure through shifting competitive interactions among different functional groups in natural or agricultural systems.

### Uncertainties

The magnitude of night temperature treatment did have an impact on most of the parameters that were investigated in the study. The negative effects of HNT and LNT on plants ecophysiological parameters (*A*_net_, *g*_s_, and tissue N), morphological parameters (SLA, LAI, and LAR), and yield related parameters (above-ground and below-ground biomass, fruit size and dry weight) were more evident with the increasing magnitude of HNT and LNT treatment (**Figures 7–9**). Consistent with the results discussed before, plant peak physiology and growth mostly occurred at night temperatures higher than the ambient, especially for leaf growth. Plant height, on the other hand, was even stimulated by HNT and not much affected by LNT. Whether plants at HNT tend to be thinner and taller requires further investigation. In all cases, photosynthesis shows an optimum temperature that roughly corresponds to the middle of the non-harmful range and drops off with an increased slope as temperatures rise above the thermal optimum.

It is essential that potential confounding factors be considered in a meta-analysis, which synthesizes results from a large number of studies conducted under a variety of growing conditions on different plant species. In our analysis, studies in plant responses to other environmental stresses (e.g., drought, low nutrients, light deficiency, or elevated ozone) were excluded. In addition to the variation caused by PFTs, different experimental design can be also responsible for the inconsistent responses (Mohammed and Tarpley, 2009a,b; Nagarajan et al., 2010; Cheesman and Klaus, 2013; Rehmani et al., 2014). Here we focused on the effects of pot size (<10L vs. >10L) and treatment duration (short vs. long term) on plant responses. Responses of plant growth under HNT and LNT may vary with time because thermal sensitivity of plants may differ among growth stages. Long-term LNT treatment had a stronger negative effect on *A*_net_ than short-term treatment, whereas HNT treatment of different durations had no significant effect on *A*_net_. On the contrary, *R*_d_ was increased greater in long-term HNT but not different between different LNT durations. Several studies had reported the long-term acclimatization of dark respiration on tropical trees (Atkin and Tjoelker, 2003; Cheesman and Klaus, 2013). Total biomass responded differently between different treatment durations of HNT and LNT, though no significant experiment duration effect was found in above- and below-ground dry matter. Plant growth and yield were decreased less at long-term HNT, which might be related to plant acclimation ability (Hare et al., 1998; Wahid et al., 2007). Pot size significantly altered the responses of *A*_net_, non-structural carbohydrates and total biomass to HNT and LNT. Small pots constrained below-ground growth along with more limitation on above-ground growth (Thomas and Strain, 1991; Loh et al., 2003; Climent et al., 2011), given that a skimpy supply of nutrients and water which might induce strong nutrient or water inhibition (Walters and Reich, 1989).

## Conclusion

We found that both HNT and LNT had a negative effect on plants yield, with the HNT effect primarily related to reduced biomass allocation to reproductive organs, flower development and seed maturation and the LNT effect more related to a negative effect on total biomass. HNT accelerated plants ecophysiological processes, including photosynthesis and dark respiration, while LNT slowed these processes. The responses to LNT and HNT varied significantly among different PFTs. HNT stimulated photosynthesis in C_3_, woody and non-crop species, but inhibited it in herbaceous, and had no effect in crops. LNT caused more reduction of *A*_net_ in woody than in herbaceous species but decreased it for both crops and non-crops with no significant difference. Both experimental settings and durations had significant effects in plants responses to night temperature change. Long-term HNT led to a relatively smaller loss of yield while the response of yield to LNT was unchanged by different treatment durations. The magnitude of night temperature did have an impact on most of the parameters that were investigated in the study. Plants peak physiology and growth mostly occurred at night temperatures higher than the ambient, especially for leaf growth. Our results suggest that the diurnal variations in vegetation responses to night temperature changes are important for understanding the changes in vegetation photosynthetic activity and growth in future climates. Such diurnal variations, however, have rarely been incorporated into current modeling studies on vegetation responses to global warming—which are overwhelmingly based on daily or growing season mean air temperature and will not capture the response of vegetation to asymmetric diurnal temperature changes. New field experiments with different elevated temperatures during day vs. night, and across different seasons, are urgently needed for different plant functional groups. Such experiments will shed new light on the ecophysiological effects of night-time temperature change during different seasons, and can be used to improve the performance of current land surface models. The functional type specific response patterns of plants to night temperature changes are critical for obtaining credible predictions of the changes in food production, carbon sequestration and climate regulation.

## Author Contributions

DW proposed the research idea. PJ collected and analyzed the data and then wrote the paper. DW, CZ, and JC help modify the paper.

## Conflict of Interest Statement

The authors declare that the research was conducted in the absence of any commercial or financial relationships that could be construed as a potential conflict of interest.

## Abbreviations

ANT: ambient night temperature
*A*_net_: net CO_2_ assimilation rate (μmol m^-2^ s^-1^)
C: carbon
*C*_i_: intercellular CO_2_ concentration (μmol mol^-1^)
ETR: electron transport rate (μmol m^-2^ s^-1^)
*F*_v_/*F*_m_: Photosystem II (PSII) efficiency
*g*_s_: stomatal conductance (mol m^-2^ s^-1^)
HNT: high night temperature
LNT: low night temperature
LAI: leaf area index
LAR: leaf area ratio (cm^2^ g^-1^)
N: nitrogen
*J*_max_: maximum electron transport rate (μmol m^-2^ s^-1^)
PFTs: plant functional types
Φ_PSII_: PSII quantum yield
*R*_d_: dark respiration rate (μmol m^-2^ s^-1^)
RuBP: ribulose-1,5-bisphosphate carboxylase
SLA: specific leaf area (cm^2^ g^-1^)
TNC: total non-structural carbohydrate (mg g^-1^)
*T*_r_: transpiration rate (mmol m^-2^ s^-1^)
*V*_cmax_: maximum carboxylation rate (μmol m^-2^ s^-1^)
