# Supplementary material for: Plant Physiological, Morphological and Yield-Related Responses to Night Temperature Changes across Different Species and Plant Functional Types
Source: Front Plant Sci. 2016 Nov 24;7:1774. doi: 10.3389/fpls.2016.01774 (PMC5121221; doi:10.3389/fpls.2016.01774)

Supplementary Material

**Plant physiological, morphological and yield-related responses to night temperature changes across different species and plant functional types**

**Panpan Jing^1^, Dan Wang^1*^, Chunwu Zhu^2^, Jiquan Chen^3*^**

^1^International Center for Ecology, Meteorology and Environment, School of Applied Meteorology, Nanjing University of Information Science and Technology, Nanjing 210044, China

^2^State Key Laboratory of Soil and Sustainable Agriculture, Institute of Soil Science, Chinese Academy of Sciences, NO.71 East Beijing Road, Nanjing 210008, China

^3^CGCEO/Geography, Michigan State University, East Lansing, MI 48823, USA

Author for correspondence:

Dan Wang, Ph.D.

Tel: 86-13062529921

E-mail: [wangdan.nuist@outlook.com](mailto:wangdan.nuist@outlook.com)

Jiquan Chen, Ph.D.

Tel: 517-884-1884

E-mail: [jqchen@msu.com](mailto:wangdan.nuist@outlook.com)

**Supplementary material S1:** The journal articles from which data were collected.

Albertine, J.M., and Manning, W.J. (2009). Elevated night soil temperatures result in earlier incidence and increased extent of foliar ozone injury to common bean (Phaseolus vulgaris L.). Environmental Pollution 157(3), 711-713. doi: 10.1016/j.envpol.2008.10.025.

Aloni, B., Pressman, E., and Karni, L. (1999). The effect of fruit load, defoliation and night temperature on the morphology of pepper flowers and on fruit shape. Annals of Botany 83(5), 529-534. doi: 10.1006/anbo.1999.0852.

Bange, M.P., and Milroy, S.P. (2004). Impact of short-term exposure to cold night temperatures on early development of cotton (Gossypium hirsutum L.). Australian Journal of Agricultural Research 55(6), 655-664. doi: 10.1071/ar03221.

Banon, S., Fernandez, J.A., Franco, J.A., Torrecillas, A., Alarcon, J.J., and Sanchez-Blanco, M.J. (2004). Effects of water stress and night temperature preconditioning on water relations and morphological and anatomical changes of Lotus creticus plants. Scientia Horticulturae 101(3), 333-342. doi: 10.1016/j.scienta.2003.11.007.

Bell, M.J., Wright, G.C., and Hammer, G.L. (1992). Night temperature affects radiation-use efficiency in peanut. Crop Science 32(6), 1329-1335.

Bertamini, M., Muthuchelian, K., Rubinigg, M., Zorer, R., and Nedunchezhian, N. (2005). Low-night temperature (LNT) induced changes of photosynthesis in grapevine (Vitis vinifera L.) plants. Plant Physiology and Biochemistry 43(7), 693-699. doi: 10.1016/j.plaphy.2005.06.001.

Bertamini, M., Muthuchelian, K., Rubinigg, M., Zorer, R., Velasco, R., and Nedunchezhian, N. (2006). Low-night temperature increased the photoinhibition of photosynthesis in grapevine (Vitis vinifera L. cv. Riesling) leaves. Environmental and Experimental Botany 57(1-2), 25-31. doi: 10.1016/j.envexpbot.2005.04.002.

Bertamini, M., Zulini, L., Muthuchelian, K., and Nedunchezhian, N. (2007). Low night temperature effects on photosynthetic performance on two grapevine genotypes. Biologia Plantarum 51(2), 381-385. doi: 10.1007/s10535-007-0080-2.

Blackshaw, R.E., and Entz, T. (1995). Day and night temperature effects on vegetative growth of Erodium cicutarium. Weed Research 35(6), 471-476. doi: 10.1111/j.1365-3180.1995.tb01644.x.

Carvalho, S.M.P., Heuvelink, E., Cascais, R., and Van Kooten, O. (2002). Effect of day and night temperature on internode and stem length in chrysanthemum: Is everything explained by DIF? Annals of Botany 90(1), 111-118. doi: 10.1093/aob/mcf154.

Cheesman, A.W., and Winter, K. (2013). Elevated night-time temperatures increase growth in seedlings of two tropical pioneer tree species. New Phytologist 197(4), 1185-1192. doi: 10.1111/nph.12098.

Chen, L., Xu, M., Zheng, Y., Men, Y., Sheng, J., and Shen, L. (2014). Growth promotion and induction of antioxidant system of tomato seedlings (Solanum lycopersicum L.) by endophyte TPs-04 under low night temperature. Scientia Horticulturae 176, 143-150. doi: 10.1016/j.scienta.2014.06.032.

Chen, W.-H., Tseng, Y.-C., Liu, Y.-C., Chuo, C.-M., Chen, P.-T., Tseng, K.-M., et al. (2008). Cool-night temperature induces spike emergence and affects photosynthetic efficiency and metabolizable carbohydrate and organic acid pools in Phalaenopsis aphrodite. Plant Cell Reports 27(10), 1667-1675. doi: 10.1007/s00299-008-0591-0.

Cheng, W., Sakai, H., Hartley, A., Yagi, K., and Hasegawa, T. (2008). Increased night temperature reduces the stimulatory effect of elevated carbon dioxide concentration on methane emission from rice paddy soil. Global Change Biology 14(3), 644-656. doi: 10.1111/j.1365-2486.2007.01532.x.

Cheng, W., Sakai, H., Yagi, K., and Hasegawa, T. (2009). Interactions of elevated CO_2_ and night temperature on rice growth and yield. Agricultural and Forest Meteorology 149(1), 51-58. doi: 10.1016/j.agrformet.2008.07.006.

Cheng, W., Sakai, H., Yagi, K., and Hasegawa, T. (2010). Combined effects of elevated CO_2_ and high night temperature on carbon assimilation, nitrogen absorption, and the allocations of C and N by rice (Oryza sativa L.). Agricultural and Forest Meteorology 150(9), 1174-1181. doi: 10.1016/j.agrformet.2010.05.001.

Darnell, R.L., Cruz-Huerta, N., and Williamson, J.G. (2013). Night temperature and source-sink effects on growth, leaf carbon exchange rate, and carbohydrate accumulation in bell pepper ovaries. Journal of the American Society for Horticultural Science 138(5), 331-337.

Deal, D.L., Raulston, J.C., and Hinesley, L.E. (1990). Leaf color retention, dark respiration, and growth of red-leafed japanese maples under high night temperatures. Journal of the American Society for Horticultural Science 115(1), 135-140.

Dejong, J., and Smeets, L. (1982). Effect of day and night temperatures during long photoperiods on the vegetative growth and flowering of chrysanthemum-morifolium ramat. Scientia Horticulturae 17(3), 271-275. doi: 10.1016/0304-4238(82)90050-4.

Doto, A.L., and Whittington, W.J. (1981). Responses of cow pea (vigna-unguiculata) varieties and their hybrids to variation in day and night temperature regimes. Annals of Applied Biology 97(2), 213-219. doi: 10.1111/j.1744-7348.1981.tb03014.x.

Fang, S.B, Ren, S., and Tan, K. (2013). Responses of winter wheat to higher night temperature in spring as compared within whole growth period by controlled experiments in North China. Journal of Food Agriculture & Environment 11(1), 777-781.

Flexas, J., Badger, M., Chow, W.S., Medrano, H., and Osmond, C.B. (1999). Analysis of the relative increase in photosynthetic O_2_ uptake when photosynthesis in grapevine leaves is inhibited following low night temperatures and/or water stress. Plant Physiology 121(2), 675-684. doi: 10.1104/pp.121.2.675.

Frantz, J.M., Cometti, N.N., and Bugbee, B. (2004). Night temperature has a minimal effect on respiration and growth in rapidly growing plants. Annals of Botany 94(1), 155-166. doi: 10.1093/aob/mch122.

Friend, D.J.C. (1981). Effect of night temperature on flowering and fruit size in pineapple (ananas-comosus l merrill. Botanical Gazette 142(2), 188-190. doi: 10.1086/337211.

Fu, J.M., and Huang, B.R. (2003). Growth and physiological response of creeping bentgrass to elevated night temperature. Hortscience 38(2), 299-301.

Gimenez, D.O., and Rumi, C.P. (1988). Interaction of night temperature and photoperiod on bromus-unioloides hbk aerial biomass components. Environmental and Experimental Botany 28(1), 1-7. doi: 10.1016/0098-8472(88)90039-1.

Hao, J.H., Li, T.L., Xu, T., Qi, H.Y., and Qi, M.F. (2010). Low night-temperatures affect the metabolism of raffinose-family oligosaccharides in melon (Cucumis melo L.) leaves during fruit expansion. Journal of Horticultural Science & Biotechnology 85(3), 260-266.

Hao, J.H., Yang, R., Fang, K.F., Wang, J.L., Zhang, Q., Shen, Y.Y., et al. (2014). Low night temperatures inhibit galactinol synthase gene expression and phloem loading in melon leaves during fruit development. Russian Journal of Plant Physiology 61(2), 178-187. doi: 10.1134/s1021443714020058.

Haynes, K.G., and Haynes, F.L. (1988). The effect of day and night temperatures on bud initiation and flowering in diploid potatoes. American Potato Journal 65(10), 589-596. doi: 10.1007/bf02908343.

Hewitt, J.D., Casey, L.L., and Zobel, R.W. (1985). Effect of day length and night temperature on starch accumulation and degradation in soybean. Annals of Botany 56(4), 513-522.

Hückstädt, A.B., Suthaparan, A., Mortensen, L.M., and Gislerød, H.R. (2013). The effect of low night and high day temperatures on photosynthesis in tomato. American Journal of Plant Sciences 04(12), 2323-2331. doi: 10.4236/ajps.2013.412288.

Ibrahim, M.A., Maenpaa, M., Hassinen, V., Kontunen-Soppela, S., Malec, L., Rousi, M., et al. (2010). Elevation of night-time temperature increases terpenoid emissions from Betula pendula and Populus tremula. Journal of Experimental Botany 61(6), 1583-1595. doi: 10.1093/jxb/erq034.

Kanno, K., Mae, T., and Makino, A. (2009). High night temperature stimulates photosynthesis, biomass production and growth during the vegetative stage of rice plants. Soil Science and Plant Nutrition 55(1), 124-131. doi: 10.1111/j.1747-0765.2008.00343.x.

Kanno, K., and Makino, A. (2010). Increased grain yield and biomass allocation in rice under cool night temperature. Soil Science and Plant Nutrition 56(3), 412-417. doi: 10.1111/j.1747-0765.2010.00473.x.

Kano, Y., and Mano, K. (2002). The effects of night soil-temperatures on diurnal changes in carbohydrate contents in roots and stems of sweet potatoes (Ipomoea batatas Poir.). Journal of the Japanese Society for Horticultural Science 71(6), 747-751.

Khayat, E., Ravad, D., and Zieslin, N. (1985). The effects of various night-temperature regimes on the vegetative growth and fruit production of tomato plants. Scientia Horticulturae 27(1-2), 9-13. doi: 10.1016/0304-4238(85)90049-4.

Khayat, E., and Zieslin, N. (1986). Effect of different night temperature regimes on the assimilation, transport and metabolism of carbon in rose plants. Physiologia Plantarum 67(4), 608-613. doi: 10.1111/j.1399-3054.1986.tb05064.x.

Kim, W.S., and Lee, J.S. (2008). Growth and light use efficiency under different light intensities of cut rose 'Rote Rose' as affected by night temperature. Horticulture Environment and Biotechnology 49(4), 226-231.

Kjaer, K.H., Hansson, I.M., Thorup-Kristensen, K., Rosenqvist, E., and Aaslyng, J.M. (2008). Root-zone heating at a night air temperature of 8 degrees C does not decrease starch accumulation in Chrysanthemum morifolium. Journal of Horticultural Science & Biotechnology 83(3), 381-387.

Kjaer, K.H., Thorup-Kristensen, K., Rosenqvist, E., and Aaslying, I.M. (2007). Low night temperatures change whole-plant physiology and increase starch accumulation in Chrysanthemum morifolium. Journal of Horticultural Science & Biotechnology 82(6), 867-874.

Kjaer, K.H., Thorup-Kristensen, K., Rosenqvist, E., and Aaslyng, J.M. (2010). Leaf carbohydrate contents and growth of chrysanthemum morifolium in response to low night temperatures and high CO_2_. European Journal of Horticultural Science 75(3), 118-122.

Langton, F.A., and Cockshull, K.E. (1997). Is stem extension determined by DIF or by absolute day and night temperatures? Scientia Horticulturae 69(3-4), 229-237. doi: 10.1016/s0304-4238(97)00020-4.

Lee, C.C., Bilderback, T.E., and Thomas, J.F. (1991). Growth-responses of heptacodium-miconioides to various photoperiods and day night temperatures. Journal of the American Society for Horticultural Science 116(4), 646-650.

Lee, C.H., and Cho, M.W. (2011). Control of unseasonable flowering in chrysanthemum 'baekma' by 2-chloroethylphosphonic acid and night temperature. Korean Journal of Horticultural Science & Technology 29(6), 539-548.

Lepage, I., Dejong, J., and Smeets, L. (1984). Effect of day and night temperatures during short photoperiods on growth and flowering of chrysanthemum-morifolium ramat. Scientia Horticulturae 22(4), 373-381.

Lin, Q., Abe, S., Nose, A., Sunami, A., and Kawamitsu, Y. (2006). Effects of high night temperature on crassulacean acid metabolism (CAM) photosynthesis of Kalanchoe pinnata and Ananas comosus. Plant Production Science 9(1), 10-19. doi: 10.1626/pps.9.10.

Liu, Y.C., Tseng, K.M., Chen, C.C., Tsai, Y.T., Liu, C.H., Chen, W.H., et al. (2013). Warm-night temperature delays spike emergence and alters carbon pool metabolism in the stem and leaves of Phalaenopsis aphroide. Scientia Horticulturae 161, 198-203. doi: 10.1016/j.scienta.2013.06.046.

Liu, Y.F., Li, T.L., Xu, T., Qi, M.F., Xu, C.Q., and Qi, H.Y. (2011). Effect of low night temperature treatment and recovery on photosynthesis and the allocation of absorbed light energy in tomato (Lycopersicon esculentum Mill.) leaves. Journal of Horticultural Science & Biotechnology 86(2), 91-96.

Liu, Y.F., Qi, M.F., and Li, T.L. (2012). Photosynthesis, photoinhibition, and antioxidant system in tomato leaves stressed by low night temperature and their subsequent recovery. Plant Science 196, 8-17. doi: 10.1016/j.plantsci.2012.07.005.

Liu, Y.F., Han, X.R., Zhan, X.M., Yang, J.F., Wang, Y.Z., Song, Q.B., et al. (2013). Regulation of calcium on peanut photosynthesis under low night temperature stress. Journal of Integrative Agriculture 12(12), 2172-2178. doi: 10.1016/s2095-3119(13)60411-6.

Liu, Y.F., Zhang, G.X., Qi, M.F., and Li, T.L. (2015). Effects of calcium on photosynthesis, antioxidant system, and chloroplast ultrastructure in tomato leaves under low night temperature stress. Journal of Plant Growth Regulation 34(2), 263-273. doi: 10.1007/s00344-014-9462-9.

Loka, D.A., and Oosterhuis, D.M. (2010). Effect of high night temperatures on cotton respiration, ATP levels and carbohydrate content. Environmental and Experimental Botany 68(3), 258-263. doi: 10.1016/j.envexpbot.2010.01.006.

Lucidos, J.G., Ryu, K.B., Younis, A., Kim, C.K., Hwang, Y.-J., Son, B.-G., et al. (2013). Different day and night temperature responses in Lilium hansonii in relation to growth and flower development. Horticulture Environment and Biotechnology 54(5), 405-411. doi: 10.1007/s13580-013-1241-1.

Malek, A.A., Blazich, F.A., Warren, S.L., and Shelton, J.E. (1992). Initial growth of seedlings of flame azalea in response to day night temperature. Journal of the American Society for Horticultural Science 117(2), 216-219.

Masuda, M., Kato, K., Murakami, K., Nakamura, H., Ojiewo, C.O., and Masinde, P.W. (2007). Partial fertility restoration as affected by night temperature in a season-dependent male-sterile mutant tomato, Lycopersicon esculentum mill. Journal of the Japanese Society for Horticultural Science 76(1), 41-46. doi: 10.2503/jjshs.76.41.

Miao, M.M., Xu, X., Chen, X., Xue, L., and Cao, B. (2007). Cucumber carbohydrate metabolism and translocation under chilling night temperature. Journal of Plant Physiology 164(5), 621-628. doi: 10.1016/j.jplph.2006.02.005.

Miao, M., Zhang, Z., Xu, X., Wang, K., Cheng, H., and Cao, B. (2009). Different mechanisms to obtain higher fruit growth rate in two cold-tolerant cucumber (Cucumis sativus L.) lines under low night temperature. Scientia Horticulturae 119(4), 357-361. doi: 10.1016/j.scienta.2008.08.028.

Mohammed, A.R., Cothren, J.T., and Tarpley, L. (2013). High night temperature and abscisic acid affect rice productivity through altered photosynthesis, respiration and spikelet fertility. Crop Science 53(6), 2603-2612. doi: 10.2135/cropsci2013.01.0060.

Mohammed, A.R., and Tarpley, L. (2010). Effects of high night temperature and spikelet position on yield-related parameters of rice (Oryza sativa L.) plants. European Journal of Agronomy 33(2), 117-123. doi: 10.1016/j.eja.2009.11.006.

Mohammed, A.R., and Tarpley, L. (2011a). Effects of night temperature, spikelet position and salicylic acid on yield and yield-related parameters of rice (oryza sativa l.) Plants. Journal of Agronomy and Crop Science 197(1), 40-49. doi: 10.1111/j.1439-037X.2010.00439.x.

Mohammed, A.R., and Tarpley, L. (2011b). High night temperature and plant growth regulator effects on spikelet sterility, grain characteristics and yield of rice (Oryza sativa L.) plants. Canadian Journal of Plant Science 91(2), 283-291. doi: 10.4141/cjps10038.

Morita, S., Yonemaru, J., and Takanashi, J. (2005). Grain growth and endosperm cell size under high night temperatures in rice (Oryza sativa L.). Annals of Botany 95(4), 695-701. doi: 10.1093/aob/mci071.

Narayanan, S., Prasad, P.V.V., Fritz, A.K., Boyle, D.L., and Gill, B.S. (2015). Impact of high night-time and high daytime temperature stress on winter wheat. Journal of Agronomy and Crop Science 201(3), 206-218. doi: 10.1111/jac.12101.

Neales, T.F., Sale, P.J.M., and Meyer, C.P. (1980). Carbon-dioxide assimilation by pineapple plants, ananas-comosus (l) merr .2. effects of variation of the day-night temperature regime. Australian Journal of Plant Physiology 7(4), 375-385.

Oda, M., Kitada, K., Ozawa, T., and Ikeda, H. (2005). Initiation and development of flower truss in 'momotaro' tomato plants associated with night temperature, and decrease in the number of leaves under the first truss by raising plug seedlings at a cool. Journal of the Japanese Society for Horticultural Science 74(1), 42-46. doi: 10.2503/jjshs.74.42.

Papadopoulos, A.P., and Hao, X.M. (2000). Effects of day and night air temperature on growth, productivity and energy use of long English cucumber. Canadian Journal of Plant Science 80(1), 143-150.

Patterson, D.T. (1990). Effects of day and night temperature on vegetative growth of texas panicum (panicum-texanum). Weed Science 38(4-5), 365-373.

Patterson, D.T. (1993). Effects of day and night temperature on goatsrue (galega-officinalis) and alfalfa (medicago-sativa) growth. Weed Science 41(1), 38-45.

Peraudeau, S., Rogues, S., Quinones, C.O., Fabre, D., Van Rie, J., Ouwerkerk, P.B.F., et al. (2015). Increase in night temperature in rice enhances respiration rate without significant impact on biomass accumulation. Field Crops Research 171, 67-78. doi: 10.1016/j.fcr.2014.11.004.

Pollet, B., Vanhaecke, L., Dambre, P., Lootens, P., and Steppe, K. (2011). Low night temperature acclimation of Phalaenopsis. Plant Cell Reports 30(6), 1125-1134. doi: 10.1007/s00299-011-1021-2.

Prasad, P.V.V., and Djanaguiraman, M. (2011). High night temperature decreases leaf photosynthesis and pollen function in grain sorghum. Functional Plant Biology 38(12), 993-1003. doi: 10.1071/fp11035.

Pressman, E., Shaked, R., and Firon, N. (2006). Exposing pepper plants to high day temperatures prevents the adverse low night temperature symptoms. Physiologia Plantarum 126(4), 618-626. doi: 10.1111/j.1399-3054.2005.00623.x.

Prieto, P., Penuelas, J., Llusia, J., Asensio, D., and Estiarte, M. (2009). Effects of long-term experimental night-time warming and drought on photosynthesis, Fv/Fm and stomatal conductance in the dominant species of a Mediterranean shrubland. Acta Physiologiae Plantarum 31(4), 729-739. doi: 10.1007/s11738-009-0285-4.

Qi, H.Y., Hua, L., Zhao, L., and Zhou, L. (2011). Carbohydrate metabolism in tomato (Lycopersicon esculentum Mill.) seedlings and yield and fruit quality as affected by low night temperature and subsequent recovery. African Journal of Biotechnology 10(30), 5743-5749.

Rahman, H.U., Hadley, P., Pearson, S., and Khan, M.J. (2013). Response of cauliflower (brassica oleracea l. Var. Botrytis) growth and development after curd initiation to different day and night temperatures. Pakistan Journal of Botany 45(2), 411-420.

Rapacz, M. (1998). The effects of day and night temperatures during early growth of winter oilseed rape (Brassica napus L var oleifera cv Gorczanski) seedlings on their morphology and cold acclimation responses. Acta Physiologiae Plantarum 20(1), 67-72. doi: 10.1007/s11738-998-0045-x.

Rehmani, M.I.A., Wei, G., Hussain, N., Ding, C., Li, G., Liu, Z., et al. (2014). Yield and quality responses of two indica rice hybrids to post-anthesis asymmetric day and night open-field warming in lower reaches of Yangtze River delta. Field Crops Research 156, 231-241. doi: 10.1016/j.fcr.2013.09.019.

Sao Pedro Machado, D.F., Machado, E.C., Machado, R.S., and Ribeiro, R.V. (2010). Effects of low night temperature and rootstocks on diurnal variation of leaf gas exchange rates and photochemical activity of 'valencia' sweet orange plants. Revista Brasileira De Fruticultura 32(2), 351-359. doi: 10.1590/s0100-29452010005000064.

Sao Pedro Machado, D.F., Magalhaes Andrade Lagoa, A.M., Ribeiro, R.V., Ribeiro Marchiori, P.E., Machado, R.S., and Machado, E.C. (2013). Low night temperature and water deficit on photosynthesis of sugarcane. Pesquisa Agropecuaria Brasileira 48(5), 487-495. doi: 10.1590/s0100-204x2013000500004.

Sao Pedro Machado, D.F., Ribeiro, R.V., Gomes da Silveira, J.A., Magalhaes Filho, J.R., and Machado, E.C. (2013). Rootstocks induce contrasting photosynthetic responses of orange plants to low night temperature without affecting the antioxidant metabolism. Theoretical and Experimental Plant Physiology 25(1), 26-35.

Schoppach, R., and Sadok, W. (2013). Transpiration sensitivities to evaporative demand and leaf areas vary with night and day warming regimes among wheat genotypes. Functional Plant Biology 40(7), 708-718. doi: 10.1071/fp13028.

Senecal, M., Dansereau, B., and Paquin, R. (1989). Fertilization and night temperature effects on growth and carbohydrate status of poinsettia. Canadian Journal of Plant Science 69(1), 347-349.

Serra, G., and Carrai, C. (1988). Effect of prolonged day temperature in night-time and different levels of night temperature on growth of Cordyline "Lord Robertson". Acta Horticulturae (229), 393-398.

Shah, F., Huang, J., Cui, K., Nie, L., Shah, T., Wu, W., et al. (2011). Physiological and biochemical changes in rice associated with high night temperature stress and their amelioration by exogenous application of ascorbic acid (vitamin C). Australian Journal of Crop Science 5(13), 1810-1816.

Shaked, R., Rosenfeld, K., and Pressman, E. (2004). The effect of low night temperatures on carbohydrates metabolism in developing pollen grains of pepper in relation to their number and functioning. Scientia Horticulturae 102(1), 29-36. doi: 10.1016/j.scienta.2003.12.007.

Shi, W.J., Muthurajan, R., Rahman, H., Selvam, J., Peng, S., Zou, Y., et al. (2013). Source-sink dynamics and proteomic reprogramming under elevated night temperature and their impact on rice yield and grain quality. New Phytologist 197(3), 825-837. doi: 10.1111/nph.12088.

Starrett, M.C., Blazich, F.A., and Warren, S.L. (1993). Initial growth of rosebay rhododendron seedlings as influenced by day and night temperatures. Hortscience 28(7), 705-707.

Teragishi, A., Kanbara, Y., and Ono, H. (2001). The effects of different night temperatures and the amount of hydroponic solution on the growth and fruiting of own-rooted fig cuttings. Journal of the Japanese Society for Horticultural Science 70(1), 1-6.

Thingnaes, E., Torre, S., Ernstsen, A., and Moe, R. (2003). Day and night temperature responses in Arabidopsis: Effects on gibberellin and auxin content, cell size, morphology and flowering time. Annals of Botany 92(4), 601-612. doi: 10.1093/aob/mcg176.

Thomas, J.F., and Raper, C.D. (1981). Day and night temperature influence on carpel initiation and growth in soybeans. Botanical Gazette 142(2), 183-187. doi: 10.1086/337210.

Thomas, J.F., Raper, C.D., and Weeks, W.W. (1981). Day and night temperature effects on nitrogen and soluble carbohydrate allocation during early reproductive growth in soybeans. Agronomy Journal 73(4), 577-582.

Tian, J.S., Hu, Y.Y., Gan, X.X., Zhang, Y.L., Hu, X.B., Gou, L., et al. (2013). Effects of increased night temperature on cellulose synthesis and the activity of sucrose metabolism enzymes in cotton fiber. Journal of Integrative Agriculture 12(6), 979-988. doi: 10.1016/s2095-3119(13)60318-4.

Tsujita, M.J. (1982). Supplemental high-pressure sodium lighting and night temperature effects on seed geraniums. Canadian Journal of Plant Science 62(1), 149-153.

Tsujita, M.J., Ormrod, D.P., and Craig, W.W. (1981). Soil heating and reduced night temperature effects on Chrysanthemums. Canadian Journal of Plant Science 61(2), 345-350.

Turner, A.D., and Ewing, E.E. (1988). Effects of photoperiod, night temperature, and irradiance on flower production in the potato. Potato Research 31(2), 257-268. doi: 10.1007/bf02365534.

Uehara, N., Sasaki, H., Aoki, N., and Ohsugi, R. (2009). Effects of the temperature lowered in the daytime and night-time on sugar accumulation in sugarcane. Plant Production Science 12(4), 420-427.

Veatch-Blohm, M.E., Ray, D.T., and Gehrels, A. (2007). Night temperature, rubber production, and carbon exchange in guayule. Industrial Crops and Products 25(1), 34-43. doi: 10.1016/j.indcrop.2006.06.019.

Verheul, M.J., Sonsteby, A., and Grimstad, S.O. (2007). Influences of day and night temperatures on flowering of Fragaria x ananassa Duch., cvs. Korona and Elsanta, at different photoperiods. Scientia Horticulturae 112(2), 200-206. doi: 10.1016/j.scienta.2006.12.022.

Warner, D.A., and Burke, J.J. (1993). Cool night temperatures alter leaf starch and photosystem-ii chlorophyll fluorescence in cotton. Agronomy Journal 85(4), 836-840.

Warner, D.A., Holaday, A.S., and Burke, J.J. (1995). Response of carbon metabolism to night temperature in cotton. Agronomy Journal 87(6), 1193-1197.

Will, R. (2000). Effect of different daytime and night-time temperature regimes on the foliar respiration of Pinus taeda: predicting the effect of variable temperature on acclimation. Journal of Experimental Botany 51(351), 1733-1739. doi: 10.1093/jexbot/51.351.1733.

Xu, Q.Z., Huang, B.G., and Wang, Z.L. (2003). Differential effects of lower day and night soil temperatures on shoot and root growth of creeping bentgrass. Hortscience 38(3), 449-454.

Yang, J., Kong, Q., and Xiang, C. (2009). Effects of low night temperature on pigments, chl a fluorescence and energy allocation in two bitter gourd (Momordica charantia L.) genotypes. Acta Physiologiae Plantarum 31(2), 285-293. doi: 10.1007/s11738-008-0231-x.

Yao, Y.L., Yamamoto, Y., Yoshida, T., Nitta, Y., and Miyazaki, A. (2000). Response of differentiated and degenerated spikelets to top-dressing, shading and day/night temperature treatments in rice cultivars with large panicles. Soil Science and Plant Nutrition 46(3), 631-641.

Yin, C.Y., Pu, X., Xiao, Q., Zhao, C., and Liu, Q. (2014). Effects of night warming on spruce root around non-growing season vary with branch order and month. Plant and Soil 380(1-2), 249-263. doi: 10.1007/s11104-014-2090-0.

Zhang, G.X., Liu, Y., Ni, Y., Meng, Z., Lu, T., and Li, T. (2014). Exogenous calcium alleviates low night temperature stress on the photosynthetic apparatus of tomato leaves. Plos One 9(5). doi: 10.1371/journal.pone.0097322.

Zhang, L., Hao, X., Li, Y., and Jiang, G. (2010). Response of greenhouse tomato to varied low pre-night temperatures at the same daily integrated temperature. Hortscience 45(11), 1654-1661.

Zhang, Y., Jiang, J., and Yang, Y.L. (2013). Acetyl salicylic acid induces stress tolerance in tomato plants grown at a low night-time temperature. Journal of Horticultural Science & Biotechnology 88(4), 490-496.

Zhang, Y.H., Li, R., and Wang, Y. (2013). Night-time warming affects N and P dynamics and productivity of winter wheat plants. Canadian Journal of Plant Science 93(3), 397-406. doi: 10.4141/cjps2012-044.

Zhu, J., Bartholomew, D.P., and Goldstein, G. (2005). “Photosynthetic gas exchange and water relations during drought in 'Smooth Cayenne' pineapple (Ananas comosus (L.) merr.) grown under ambient and elevated CO_2_ and three day/night temperatures,” in Proceedings of the Ivth International Pineapple Symposium, ed. A.R. Martinez.), 161-173.

Zieslin, N., Khayat, E., and Mor, Y. (1986). The response of rose plants to different night-temperature regimes. Acta Horticulturae (189), 181-187.

Ziska, L.H., and Manalo, P.A. (1996). "Increasing night temperature can reduce seed set and potential yield of tropical rice", in: Australian Journal of Plant Physiology.

**Supplementary Figure S2.** The frequency diagram of night temperature treatment used in the studies where the data were collected.


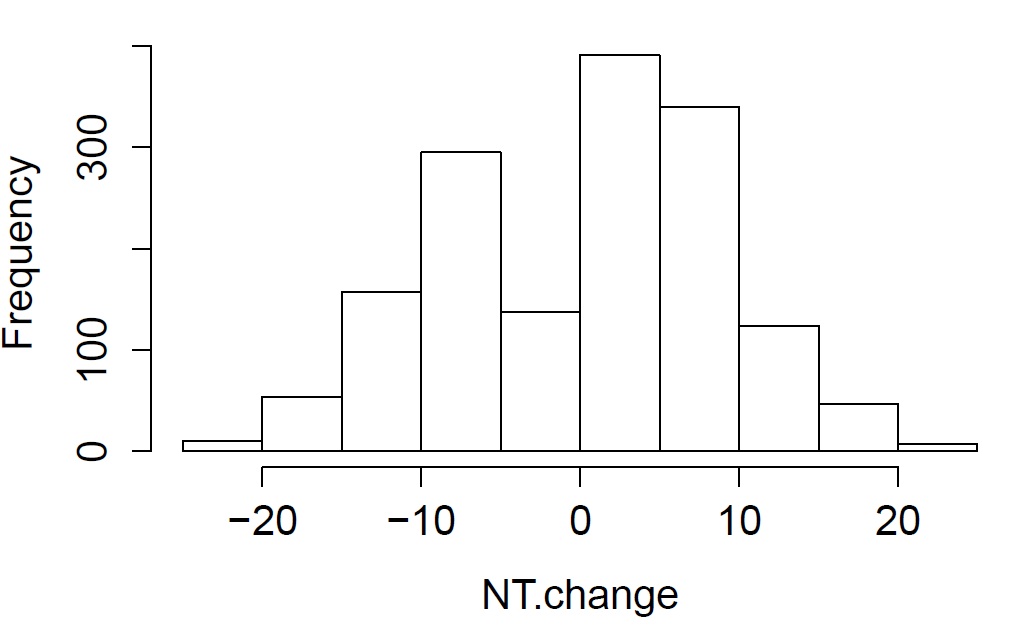


**Supplementary Table S3.** List of species, plant functional types (photosynthetic pathway, growth form and domestication) and associated references used in this study

| Species（common name with cultivar information) | Plant functional types | References |
| --- | --- | --- |
| *Acer Palmatum* Thunb.(Japanese maple) | C_3_ wood wild | Deal D.L. et al. 1990 |
| *Agrotis palustris* L.(Creeping bentgrass:'Penncross') | C_3_ herb wild | Xu Q.Z. et al. 2003; Fu J.M. and Huang B.R. 2003 |
| *Ananas comosus* L. Merr.(Pineapple: 'Smmoth Cayenne Champaka 153') | CAM herb crop | Friend D.J.C. 1981 |
| *Ananas comosus* L. Merr.(Pineapple: 'Smmoth Cayenne Champaka F-153') | CAM herb crop | Zhu J. et al. 2005 |
| *Ananas comosus*(Pineapple:'Cayenne') | CAM herb crop | Neales T.F. et al. 1980; |
| *Ananas comosus*(Pineapple:'Smmoth Cayenne N67-10') | CAM herb crop | Lin Q. et al. 2006 |
| *Arabidopsis thaliana* L*er* | C_3_ herb wild | Thingnaes E. et al. 2003 |
| Arachis hypogaea L.(Peanut:'Fenghua 1') | C_3_ herb crop | Liu Y.F. et al. 2013 |
| *Arachis hypogaea* L.(Peanut:'TMV2','Early Bunch','Virginia Bunch') | C_3_ herb crop | Bell M.J. 1992 |
| *Betula pendula*(Silver birch ) | C_3_ wood wild | Ibrahim et al. 2010 |
| *Brassica napus* L.（Oilseed rape：'Gorczanski'） | C_3_ herb crop | Rapacz M. 1998 |
| *Brassica oleracea* L.(Cauliflower) | C_3_ herb crop | Rahman et al. 2013 |
| *Bromus unioloides* | C_3_ herb wild | Gimenez D.O. and Rumi C.P. 1988 |
| *Capsicum annuum* L.(Bell pepper) | C_3_ herb crop | Darnell et al. 2013;Aloni B. et al.1999 |
| Capsicuum annuum L.(Pepper:'Fiesta','Selica') | C_3_ herb crop | Pressman E. et al. 2006 |
| *Capsicuum annuum* L.(Pepper:'Fiesta','Selica','Devilla','Mitla') | C_3_ herb crop | Shaked R. et al. 2004 |
| Chrysanthemum morifolium Ramat. (Chrysanthemum:'Accent','Boston','Lapana','Pink Gin','Rewilo','White Spider') | C_3_ herb wild | Lepage I. et al. 1984 |
| *Chrysanthemum morifolium* Ramat. (Chrysanthemum:'Promenade','Improved Mefo','Goldburst Mefo') | C_3_ herb wild | Tsujita M.J. et al. 1981 |
| *Chrysanthemum morifolium* Ramat.(Chrysanthemum) | C_3_ herb wild | Dejong J. and Smeets L. 1982 |
| *Chrysanthemum morifolium*(Chrysanthemum:'Baekma') | C_3_ herb wild | Lee C.H. and Cho M.W. 2011 |
| *Chrysanthemum morifolium*(Chrysanthemum:'Bright Golden Anne') | C_3_ herb wild | Langton F.A. and Cockshull K.E. 1997 |
| *Chrysanthemum morifolium*(Chrysanthemum:'Coral Charm') | C_3_ herb wild | Kjaer K.H. et al. 2007,2008,2010 |
| *Chrysanthemum morifolium*(Chrysanthemum:'Reagan Improved') | C_3_ herb wild | Carvalho S.M.P. et al. 2002 |
| *Citrus sinensis* L.(Orange) | C_3_ wood crop | Sao P.M. et al. 2010,2013 |
| *Cordyline terminalis*(Hawaiian Ti:'Lord Robertson') | CAM wood wild | Serra G. and Carrai C. 1988 |
| *Cucumis melo* L.(Melon:'Yumeiren') | C_3_ herb crop | Hao J.H et al. 2010,2014 |
| *Cucumis sativus* L.(Cucumber:'Corona','Aramon') | C_3_ herb crop | Papadopoulos A.P. and Hao X.M. 2000 |
| *Cucumis sativus* L.(Cucumber:'Jinyan4','NY-1') | C_3_ herb crop | Miao M.M. et al. 2007 |
| *Cucumis sativus* L.(Cucumber:'Jinyan4','NY-1','XC-1') | C_3_ herb crop | Miao M. et al. 2009 |
| *Erica multiflora L.* | C_3_ wood wild | Prieto et al. 2009 |
| *Erodium cicutarium* | C_3_ herb wild | Blackshaw R.E. and Entz T. 1995 |
| *Euphorbia pulcherrima*(Poinsettia:'Annette Hegg Dark Red') | C_3_ wood wild | Senecal M. et al. 1989 |
| *Ficus carica*(Fig:'Masui-Dauphine') | C_3_ wood crop | Teragishi et al. 2001 |
| *Ficus insipida*(Moraceae) | C_3_ wood crop | Cheesman et al. 2013 |
| *Fragaria x ananassa*(Strawberry:'Korona','Elsanta') | C_3_ herb crop | Verheul M.J. et al. 2007 |
| *Galega officinalis* L.(Goatsrue) | C_3_ herb wild | Patterson D.T. 1993 |
| *Globularia alypum L.* | C_3_ wood wild | Prieto et al. 2009 |
| *Glycine max* L. Merr.(Soybean:'Chippewa 64') | C_3_ herb crop | Hewitt J.D. et al. 1985 |
| *Glycine max* L. Merr.(Soybean:'Hoyt') | C_3_ herb crop | Frantz J.M. et al. 2004 |
| *Glycine max* L. Merr.(Soybean:'Ransom') | C_3_ herb crop | Thomas J.F. and Raper C.D. 1981;Thomas J.F. et al. 1981 |
| *Gossypium hirsutum* L.(Cotton:'DP444BG/RR') | C_3_ herb crop | Loka D.A. and Oosterhuis D.M. 2010; |
| *Gossypium hirsutum* L.(Cotton:'Paymaster HS-26') | C_3_ herb crop | Warner D.A. et al. 1995 |
| *Gossypium hirsutum* L.(Cotton:'Sicala V-2i') | C_3_ herb crop | Bange M.P. and Milroy S.P. 2004 |
| *Gossypium hirsutum* L.(Cotton:'T25','Paymaster HS-26') | C_3_ herb crop | Warner D.A. and Burke J.J. 1993 |
| *Gossypium hirsutum* L.(Cotton:'Xinluzao 13','Xinluzao 33' ) | C_3_ herb crop | Tian J.S. et al. 2013 |
| *Heptacodium miconioides* | C_3_ wood wild | Lee C.C. et al. 1991 |
| *Ipomoea batatas* Poir.(Sweet potato:'Kokei No.14') | C_3_ herb crop | Kano Y. and Mano K. 2002 |
| *Kalanchoe pinnata* | CAM herb wild | Lin Q. et al. 2006 |
| *Latuca sativa*(Lettuce:'Grand Rapids') | C_3_ herb crop | Frantz J.M. et al. 2004 |
| *Lilium hansonii* | CAM herb wild | Lucidos et al. 2013 |
| *Lotus creticus* sub.*creticus* | C_3_ herb wild | Banon S. et al. 2004 |
| *Lycopersicon esculentum* Mill.(Tomato:'Bigdena','Quest','Clarance','Conchita') | C_3_ herb crop | Zhang L. et al. 2010 |
| *Lycopersicon esculentum* Mill.(Tomato:'Liaoyuanduoli') | C_3_ herb crop | Liu Y.F. et al. 2011,2012,2015;Zhang G.X. et al. 2014; Qi H.Y. et al. 2011;Zhang Y. et al. 2013 |
| *Lycopersicon esculentum* Mill.(Tomato:'Momotaro') | C_3_ herb crop | Oda M. et al. 2005 |
| *Lycopersicon esculentum* Mill.(Tomato:'Moneymaker') | C_3_ herb crop | Langton F.A. and Cockshull K.E. 1997 |
| *Lycopersicon esculentum* Mill.(Tomato:'Moneymaker','Cherry 688') | C_3_ herb crop | Khayat E. et al. 1985 |
| *Lycopersicon esculentum* Mill.(Tomato:'Red Robin') | C_3_ herb crop | Frantz J.M. et al. 2004 |
| *Lycopersicon esculentum* Mill.(Tomato:'T-4') | C_3_ herb crop | Masuda et al. 2007 |
| *Medicago sativa* L.(Alfalfa) | C_3_ herb wild | Patterson D.T. 1993 |
| *Momordica charantia* L.(Bitter gourd) | C_3_ herb crop | Yang J. et al. 2009 |
| *Ochroma pyramidale*(Bombacaceae) | C_3_ wood wild | Cheesman et al. 2013 |
| *Oryza sativa* L. *aus*(Rice:'Kasalath','N22') | C_3_ herb crop | Peraudeau et al. 2015 |
| *Oryza sativa* L. *indica*(Rice:'Hybrid1','Hybrid2','Hybrid3','jamajigi') | C_3_ herb crop | Peraudeau et al. 2015 |
| *Oryza sativa* L. *indica*(Rice:'IR72') | C_3_ herb crop | Cheng W.G. et al. 2008,2009,2010；Ziska L.H. and Manalo P.A. 1996 |
| *Oryza sativa* L. *indica*(Rice:'Kasalath') | C_3_ herb crop | Shah et al. 2011 |
| *Oryza sativa* L. *indica*(Rice:'Teyou-559','Shanyou-63') | C_3_ herb crop | Rehmani et al. 2014 |
| *Oryza sativa* L. *indica*(Rice:'Yangdao 4') | C_3_ herb crop | Yao Y.L. et al. 2000 |
| *Oryza sativa* L. *japonica*(Rice:'Cocodrie','M202') | C_3_ herb crop | Peraudeau et al. 2015 |
| *Oryza sativa* L. *japonica*(Rice:'Nipponbare') | C_3_ herb crop | Shah et al. 2011 |
| *Oryza sativa* L. *japonica-indica*(Rice:'Akenohoshi') | C_3_ herb crop | Yao Y.L. et al. 2000 |
| *Oryza sativa* L.(Rice:'Akita-63') | C_3_ herb crop | Kanno et al. 2010 |
| *Oryza sativa* L.(Rice:'Cocodrie') | C_3_ herb crop | Mohammed A.R. and Tarpley L. 2010,2011a,2011b |
| *Oryza sativa* L.(Rice:'Kinuhikari') | C_3_ herb crop | Morita S. et al. 2005 |
| *Oryza sativa* L.(Rice:'N22','Gharib') | C_3_ herb crop | Shi W.J. et al. 2013 |
| *Oryza sativa* L.(Rice:'Notohikari') | C_3_ herb crop | Kanno et al. 2009 |
| *Oryza sativa* L.(Rice:'XL723') | C_3_ herb crop | Mohammed A.R. et al. 2013 |
| *Panicum texanum*(Texas Panicum) | C_4_ herb crop | Patterson D.T. 1990 |
| *Parthenium argentatum* Gray(Guayule) | C_3_ herb wild | Veatch-Blohm M.E. et al. 2007 |
| *Pelargonium x hortorum* Bailey(Geranium:'Fire Flash','Encounter Red','Sprinter Salmon') | C_3_ herb wild | Tsujita M.J. 1982 |
| *Phalaenopsis aphrodite* subsp. *formosana* | CAM herb wild | Liu Y.C. et al. 2013 |
| *Phalaenopsis aphrodite* subsp. *formosana*('Atlantis’) | CAM herb wild | Pollet et al. 2011 |
| *Phalaenopsis aphrodite* subsp. *Formosana*('TS97') | CAM herb wild | Chen W.H. et al. 2008 |
| *Phaseolus vulgaris* L.(Bean) | C_3_ herb crop | Albertine et al. 2009 |
| *Picea asperata* | C_3_ wood wild | Yin C.Y. et al. 2014 |
| *Pinus halepensis* L. | C_3_ wood wild | Prieto et al. 2009 |
| *Pinus taeda* L.(Loblolly pine) | C_3_ wood wild | Will R. 2000 |
| *Populus tremula*(European aspen) | C_3_ wood wild | Ibrahim et al. 2010 |
| *Rhododendron calendulaceum* Michx. Torr(Flame azalea) | C_3_ wood wild | Malek A.A. et al. 1992 |
| *Rhododendron maximum* L.(Rosebay rhododendron) | C_3_ wood wild | Starrett M.C. et al. 1993 |
| *Rosa hybrida*（Rose：'Mercedes','Sonia','Visa','Golden Times') | C_3_ wood wild | Zieslin N. et al. 1986 |
| *Rosa hybrida*（Rose：'Rote Rose') | C_3_ wood wild | Kim W.S. and Lee J.S. 2008 |
| *Rosa hybrida*（Rose：'Sonia','Golden Times') | C_3_ wood wild | Khayat E. and Zieslin N. 1986 |
| *Saccharum officinarum* L. (Sugarcane:'IACSP94-2094') | C_4_ herb crop | Sao P.M. et al. 2013 |
| *Saccharum officinarum* L. (Sugarcane:'NiF8') | C_4_ herb crop | Uehara et al. 2009 |
| *Solanum lycopersicum* L.(Tomato:'Lichun') | C_3_ herb crop | Chen L. et al. 2014 |
| *Solanum lycopersicum* L.(Tomato:'Mecano') | C_3_ herb crop | Hückstädt A.B. et al. 2013 |
| *Solanum tuberosum* L.(Potato:'DM3-1','DM5-12','DM56-4','DM66-4','DM91-5','DM27-8','DM80-12','HT75P9-31') | C_3_ herb crop | Haynes K.G. and Haynes F.L. 1988 |
| *Solanum tuberosum* L.(Potato:'DTO-33','LT-2') | C_3_ herb crop | Turner A.D. and Ewing E.E. 1988 |
| *Sorghum bicolor* L. Moench(Sorghum:'DK-28E') | C_4_ herb crop | Prasad P.V.V. et al. 2011 |
| *Triticum aestivum* L.(Wheat:'Berkut','Drysdale','Excalibur','Gladius','Krichauff','Kukri','RAC876','Sokoll') | C_3_ herb crop | Schoppach et al. 2013 |
| *Triticum aestivum* L.(Winter wheat:'Karl 92','Ventnor') | C_3_ herb crop | Narayanan S. et al. 2015 |
| *Triticum aestivum* L.(Winter wheat:'Ningmai No.15') | C_3_ herb crop | Zhang Y.H. et al. 2013; ; |
| *Triticum aestivum* L.(Winter wheat:'Super-626') | C_3_ herb crop | Fang S.B. et al. 2013 |
| *Triticum durum* Desf.(Wheat:'Karur','Miradoux','Pescadou') | C_3_ herb crop | Schoppach et al. 2013 |
| *Vigna unguiculata*(Cowpea:'SVS 98','VH 17','New Era','Tvu 1190') | C_3_ herb crop | Doto A.L. and Whittington W.J. 1981 |
| *Vitis vinifera* L.(Grapevine) | C_3_ wood crop | Bertamini M. et al. 2005 |
| Vitis vinifera L.(Grapevine:'Chardonnay','Riesling','Gordot') | C_3_ wood crop | Flexas J. et al. 1999 |
| Vitis vinifera L.(Grapevine:'Müller-Thurgau','Lagrein') | C_3_ wood crop | Bertamini M. et al. 2007 |
| *Vitis vinifera* L.(Grapevine:'Riesling') | C_3_ wood crop | Bertamini M. et al. 2006 |

**Supplementary Figure S4.** Plant physiological, morphological and yield-related responses to LNT in in C_3_ (circles), C_4_ (triangles) and CAM (stars) species. Each data point represents the mean ± 95% confidence interval (CI; whiskers). The number of observations for each variable is presented on the right of the graph.


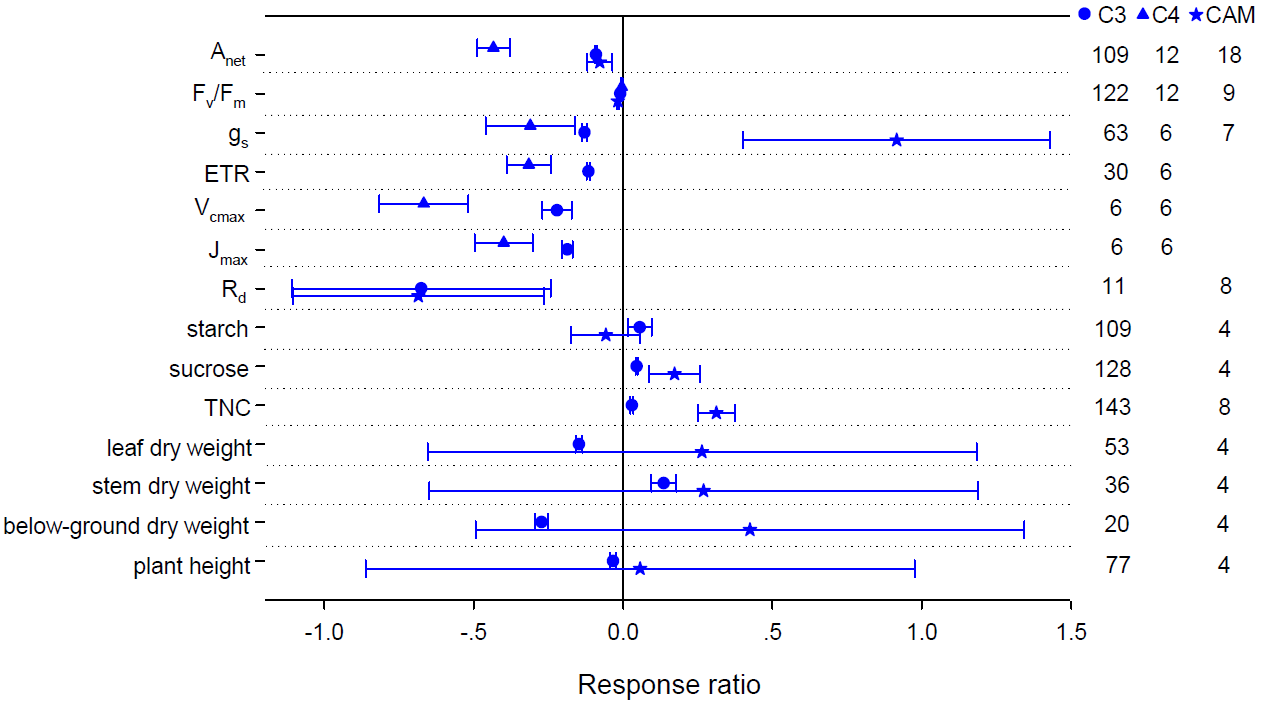

Supplement: Supplementary file 1 [file Data_Sheet_1.DOCX]
